# Comprehensive pan‐cancer analysis of mitochondrial outer membrane permeabilisation activity reveals positive immunomodulation and assists in identifying potential therapeutic targets for immunotherapy resistance

**DOI:** 10.1002/ctm2.1735

**Published:** 2024-06-20

**Authors:** Qingshan Chen, Fenglin Gao, Junwan Wu, Kaiming Zhang, Tian Du, Yuhong Chen, Ruizhao Cai, Dechang Zhao, Rong Deng, Jun Tang

**Affiliations:** ^1^ State Key Laboratory of Oncology in South China, Guangdong Provincial Clinical Research Center for Cancer Sun Yat‐sen University Cancer Center Guangzhou China; ^2^ Department of Breast Oncology Sun Yat‐sen University Cancer Center Guangzhou China; ^3^ Department of Respiratory and Critical Care Medicine The Second Affiliated Hospital of Nanjing Medical University Nanjing China; ^4^ Biotherapy Center, State Key Laboratory of Oncology in South China, Guangdong Provincial Clinical Research Center for Cancer Sun Yat‐sen University Cancer Center Guangzhou China

**Keywords:** immune resistance, immunotherapy, mitochondrial outer membrane permeabilisation, pan‐cancer

## Abstract

**Background:**

Mitochondrial outer membrane permeabilisation (MOMP) plays a pivotal role in cellular death and immune activation. A deeper understanding of the impact of tumour MOMP on immunity will aid in guiding more effective immunotherapeutic strategies.

**Methods:**

A comprehensive pan‐cancer dataset comprising 30 cancer‐type transcriptomic cohorts, 20 immunotherapy transcriptomic cohorts and three immunotherapy scRNA‐seq datasets was collected and analysed to determine the influence of tumour MOMP activity on clinical prognosis, immune infiltration and immunotherapy effectiveness. Leveraging 65 scRNA‐Seq datasets, the MOMP signature (MOMP.Sig) was developed to accurately reflect tumour MOMP activity. The clinical predictive value of MOMP.Sig was explored through machine learning models. Integration of the MOMP.Sig model and a pan‐cancer immunotherapy CRISPR screen further investigated potential targets to overcome immunotherapy resistance, which subsequently underwent clinical validation.

**Results:**

Our research revealed that elevated MOMP activity reduces mortality risk in cancer patients, drives the formation of an anti‐tumour immune environment and enhances the response to immunotherapy. This finding emphasises the potential clinical application value of MOMP activity in immunotherapy. MOMP.Sig, offering a more precise indicator of tumour cell MOMP activity, demonstrated outstanding predictive efficacy in machine‐learning models. Moreover, with the assistance of the MOMP.Sig model, FOXO1 was identified as a core modulator that promotes immune resistance. Finally, these findings were successfully validated in clinical immunotherapy cohorts of skin cutaneous melanoma and triple‐negative breast cancer patients.

**Conclusions:**

This study enhances our understanding of MOMP activity in immune modulation, providing valuable insights for more effective immunotherapeutic strategies across diverse tumours.

## Graphical abstract

MOMP activity exhibits a positive role in promoting anti‐tumour TIME and immunotherapy response. The MOMP.Sig model has a better prediction efficiency than 16 other pan‐cancer ICI signatures. MOMP.Sig model integrates pan‐cancer ICI CRISPR screen to identify key molecule FOXO1 for immunotherapy resistance. Multiple ICI clinical cohorts have verified the immune resistance effect of FOXO1.

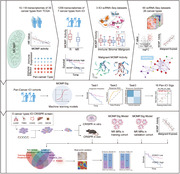

## 1 INTRODUCTION

Tumour immunotherapy using immune checkpoint inhibitors (ICIs), which activate the tumour immune microenvironment (TIME) and promote the elimination of tumour cells, has fundamentally transformed the therapeutic landscape, yielding meaningful clinical responses across various tumour types in both adjuvant and metastatic settings.^1^, ^2^ In fact, this approach has limitations, as clinical benefits can only be achieved within a specific patient subgroup. The precise identification of patients eligible for immunotherapy and the development of combination strategies to combat immune resistance are crucial unmet clinical needs.^3^, ^4^, ^5^

The well‐recognised Warburg effect in malignant cells leads to mitochondrial stress, resulting in significant reorganisation, such as lipid transfer, cristae remodelling, fragmentation and mitochondrial outer membrane permeabilisation (MOMP).^6^, ^7^ Although the role of many of these alterations is not fully understood, extensive MOMP in the mitochondrial network is recognised as a pivotal signalling centre for both immune responses and cell death pathways.^8^, ^9^ MOMP triggers the release of soluble intermembrane space proteins into the cytosol, promoting the formation of apoptosomes and the activation of caspase‐9. Active caspase‐9 subsequently leads to the cleavage and activation of caspase‐3 and caspase‐7, culminating in swift cellular demolition.^10^ In turn, apoptotic caspase‐3 cleaves gasdermin E (GSDME), inducing pyroptosis.^11^ Moreover, active GSDME facilitates MOMP, establishing a feed‐forward mechanism for caspase‐3/‐7 activation.^12^, ^13^ Additionally, the leakage of cellular contents into the cytosol due to MOMP has implications beyond triggering cell death, including the initiation of proinflammatory signalling. For instance, the release of mitochondrial DNA (mtDNA) into the cytosol is sensed by the cyclic GMP–AMP synthase (cGAS)/stimulator of interferon genes (STING) pathway, which activates innate immune signalling.^14^ Cytosolic second mitochondria‐derived activator of caspase also activates the NF‐kB signalling pathway.^15^ Elevated levels of mitochondrial reactive oxygen species resulting from MOMP are detected by NLR proteins (NLRP1, NLRP3 and NLRC4), leading to NLRP3 inflammasome activation in cells^16^ and subsequently generating proinflammatory signals and activating gasdermin D to induce pyroptosis.^17^ These findings strongly indicate that MOMP within tumours is pivotal for orchestrating cell death and immune responses governed by mitochondria. However, a detailed summary of the relationship between MOMP and immunotherapy is lacking, and there is limited detailed functional research on the role of MOMP in immunotherapy resistance.

Given the complexity and diversity of MOMP, there is currently no unified and precise experimental method to measure its extent accurately.^18^, ^19^, ^20^, ^21^, ^22^, ^23^ Additionally, the available detection methods are limited primarily to the cellular level. Moreover, in clinical oncology applications, the rapid cell death of cells in ex vivo tumour tissues obtained from tumour biopsies or surgeries makes it challenging to accurately assess the original level of MOMP in the tumour tissue. To better characterise the level of MOMP in tumour tissues, based on previous research methods,^24^, ^25^, ^26^, ^27^ we integrated the effector pathways directly activated by MOMP (apoptosis, necroptosis, cGAS/STING, NF‐kB and inflammasome pathways) to calculate MOMP activity in tumour tissues. This will aid in our deeper exploration of the correlation between tumour MOMP and immunotherapy.

In our study, we performed a comprehensive analysis of MOMP activity from a pan‐cancer bulk RNA‐Seq dataset (10 118 patients; 30 cancer types). Our analysis revealed a positive correlation between MOMP activity and the anti‐tumour TIME, indicating that increased MOMP activity positively contributes to immunotherapy responses. Further detailed investigations across three immunotherapy scRNA‐Seq datasets highlighted a significant correlation between immunotherapy response and increased MOMP activity, specifically in tumour cells, rather than in non‐tumour cells. To eliminate non‐tumour cell interference and achieve a deeper understanding of the influence of tumour cell MOMP activity on immunotherapy efficacy, we integrated pan‐cancer scRNA‐Seq datasets (65 datasets; 26 cancer types) to construct a tumour cell‐associated MOMP activity signature (MOMP.Sig). This signature exhibited excellent predictive effects in a pan‐cancer ICI cohort (1256 patients; seven cancer types) using a machine learning‐based multitask model. Subsequent efforts aimed to identify key molecules associated with immune resistance and enhance immune efficacy. We extracted genes that exhibited a negative correlation with tumour cell MOMP activity from pan‐cancer scRNA‐Seq datasets. Using the Algorithm for the Reconstruction of Accurate Cellular Networks with Adaptive Partitioning (ARACNe‐AP)^28^ and the Virtual Proteomics by Enriched Regulon Analysis (VIPER)^29^ algorithms, we compared accurately predicted immunotherapy responders and nonresponders to identify master regulators (MRs) associated with immune resistance. Furthermore, our analysis, complemented by a pan‐cancer immunotherapy CRISPR screen (five cancer types), pinpointed FOXO1 as a key MR involved in immune resistance. The role of FOXO1 was additionally confirmed in two independent immunotherapy clinical cohorts. Our study emphasises the potential of the tumour MOMP for predicting the ICI response and guiding immunotherapy strategies.

## 2 MATERIALS AND METHODS

### 2.1 Pan‐cancer data collection and processing

Pan‐cancer transcriptomic data and corresponding clinical information for 30 solid cancer types from The Cancer Genome Atlas (TCGA) were obtained by using the UCSC Xena browser (https://xena.ucsc.edu/). Pan‐cancer tumour mutation burden (TMB) and intra‐tumour heterogeneity (ITH) data were retrieved from TCGABiolinks.^30^ The homologous recombination repair deficiency (HRD) score and tumour purity for the pan‐cancer dataset were obtained from https://github.com/GerkeLab/TCGAhrd. Additionally, the pan‐cancer landscape of microsatellite instability (MSI) was derived from Bonneville et al.^31^

### 2.2 Individualised MOMP activity algorithm

**FIGURE 1:**
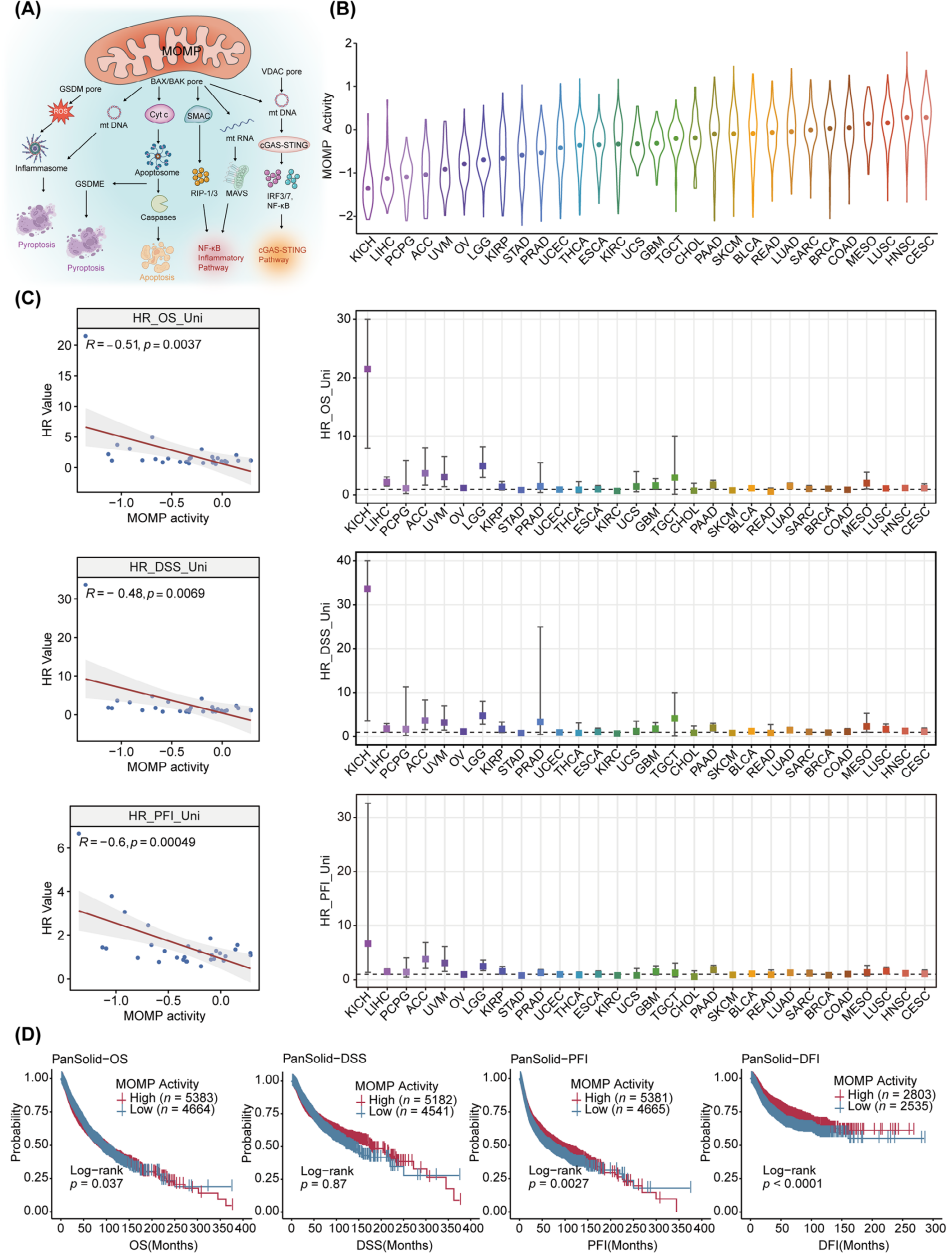
MOMP activity and survival across cancers. (A) Cellular inflammation and death signalling pathways induced by MOMP. (B) Differential MOMP activity across 30 solid cancers in TCGA, with cancer types ranked based on the average MOMP activity. (C) Linear regression analysis quantified the relationship between the average MOMP activity in 30 solid cancer types from TCGA and the corresponding HR for survival (left). Univariate Cox regression analysis revealed the influence of tumour MOMP activity on the HR (±95% CI) associated with survival, with cancer types ranked based on the average MOMP activity (right). (D) Kaplan‒Meier plot of the effect of MOMP activity on OS, DSS, PFI and DFI in the pan‐cancer cohort from TCGA. The MOMP activity level cutoff point was set at the average MOMP activity. DFI, disease‐free interval; DSS, disease‐specific survival; MOMP, mitochondrial outer membrane permeabilisation; OS, overall survival; PFI, progression‐free interval; TCGA, The Cancer Genome Atlas.

Signalling pathways indicative of MOMP activity were extracted from the Canonical pathways collection in MSigDB C2. These pathways include the apoptosis, pyroptosis, cGAS/STING, inflammasome and NF‐kB signalling pathways (Figure 1A and Table S1). These pathways constitute the MOMP activity gene set, and the MOMP activity in individual samples was calculated using the single‐sample Mann–Whitney–Wilcoxon gene set test (ssMWW–GST) method.^24^ Researchers have utilised a similar approach in various studies. For instance, in assessing the degree of T‐cell exhaustion, authors created a score integrating pathways such as interferon‐gamma, interleukin‐2, cytotoxic potential and tumour necrosis factor pathways.^27^ Similarly, in studying neutrophil extracellular trap activity, they developed a score based on genes involved in neutrophil extracellular trap formation.^26^ Recently, researchers constructed a scoring system based on six core pathways related to immunotherapy to prioritise candidate compounds for enhancing immunotherapy efficacy.^25^

### 2.3 Assessment of the TIME

The fractions of diverse cellular components in the TIME were initially calculated via the CIBERSORT tool (https://cibersortx.stanford.edu), and the TIME score was assessed via the ESTIMATE algorithm.^32^, ^33^ Subsequently, Pearson correlation analysis was conducted on the average MOMP activity of different cancer types, the average fractions of diverse cellular components and the average TIME scores. Furthermore, considering the influence of tumour purity on the TIME, patients were categorised into high and low groups based on the average tumour purity level, and Pearson correlation analysis was performed on the average MOMP activity and average TIME scores of different cancer types. For a more comprehensive understanding of the association between MOMP activity and the TIME, immune‐related molecules – encompassing antigen presentation‐related molecules, immune stimulatory molecules, immune inhibitory molecules, immune effector and suppressor cells and the immune phenotype score (IPS) – were acquired from the TCIA database (https://tcia.at/).^34^ These components were analysed for their correlation with MOMP activity.

### 2.4 Pan‐cancer ICI RNA‐Seq datasets

To confirm the predictive efficacy of MOMP activity and MOMP.Sig in immunotherapy, comprehensive transcriptomic and clinical data from various immunotherapy cohorts were collected (Table S2). Specifically, three skin cutaneous melanoma (SKCM) ICI cohorts (Hugo 2016,^35^ Riza 2017,^36^ Auslander 2018^37^) were retrieved from the GEO database with accession numbers GSE78220, GSE91061 and GSE115821. Additionally, two SKCM ICI cohorts (Van Allen 2015,^38^ Liu 2019^39^) were obtained from the dbGaP database with accession numbers phs000452.v2.p1 and phs000452.v3.p1. Furthermore, data for another SKCM ICI cohort (Gide 2019^40^) were sourced from the European Bioinformatics Institute (EBI) database with accession number PRJEB23709. Two non‐small cell lung cancer (NSCLC) ICI datasets (Jung 2021,^41^ Cho 2020^42^) were downloaded from the GEO database using the accession numbers GSE135222 and GSE126044. Two urothelial carcinoma (UC) ICI cohorts (Mariathasan 2018,^43^ Rose 2021^44^) were downloaded from the EBI and GEO databases, with accession numbers EGAS00001002556 and GSE176307, respectively. For the renal cell carcinoma (RCC) ICI cohort (Maria 2016,^45^ Braun 2020^46^), the Braun 2020 cohort was obtained from the supplementary materials provided by Braun et al.,^46^ whereas the Maria 2016 cohort was downloaded from the GEO database. In addition, data for a glioblastoma multiforme (GBM) ICI cohort (Zhao 2019^47^) were downloaded from the EBI database under accession number PRJNA482620, and data for a breast cancer (BRCA) ICI cohort (Wolf 2022^48^) were obtained from the GEO database using accession number GSE194040. Finally, a stomach adenocarcinoma (STAD) ICI cohort (Seung 2018^49^) was downloaded from the EBI database with accession number PRJEB5780. The primary clinical endpoint assessed was the objective response rate, which was evaluated in all cohorts using Response Evaluation Criteria in Solid Tumors version 1.1,^50^ except for the Hugo 2016 SKCM cohort,^35^ which used immune‐related RECIST for evaluation. Patients were divided into two categories according to their response status: complete response (CR) and partial response (PR) as responders and stable disease (SD) and progressive disease (PD) as non‐responders (Table S2).

### 2.5 ICI scRNA‐Seq datasets

The pre‐immunotherapy SKCM and BCC scRNA‐Seq datasets were acquired from the GEO database (GSE115978 and GSE123831). Quality control procedures were applied using default parameters in Omicverse (https://github.com/Starlitnightly/omicverse.git), which involved filtering out genes with low expression and low‐quality cells. Mitochondrial and ribosomal genes were also filtered from the datasets. Subsequently, the data were standardised, highly variable genes were obtained and dimensionality reduction clustering was performed. Marker genes representing various cell types from the CellMarker^51^ database were used for cell annotation (Table S3). The NSCLC scRNA‐Seq ICI dataset was sourced from GSE207422 in the GEO database. Quality control procedures were applied using default parameters in Omicverse with the exclusion of mitochondrial and ribosomal genes. Subsequent steps involved obtaining highly variable genes, reducing dimensionality, performing clustering analysis and annotating distinct cell populations. Further analysis focused on epithelial cells, stromal cells and fibroblasts. Using the infercnvpy (https://github.com/icbi‐lab/infercnvpy.git) package and employing stromal and fibroblasts as controls, the copy number variation (CNV) scores of epithelial cells were analysed, and epithelial cells exhibiting high CNV scores were categorised as tumour cells. Finally, marker genes representing various cell types from the CellMarker^51^ database were used for cell annotation (Table S3).

### 2.6 Pan‐cancer scRNA‑Seq datasets

To accurately represent MOMP activity in tumour cells, 65 scRNA‐Seq datasets covering 26 solid cancer types across 15 distinct anatomical locations were acquired from the TISCH2 portal (Table S4) to construct the MOMP.Sig. These datasets underwent dimensional reduction, clustering and cell annotation. The FindAllMarkers function from Seurat was used to identify highly expressed tumour cell‐specific genes in each single‐cell dataset (*p* value < .05 and log fold change (logFC) > 1). Subsequently, Pearson correlation analysis was employed to examine the relationship between tumour cell MOMP activity and gene expression levels. Genes closely related to MOMP activity were screened using the criteria of a *p* value < .05 and a correlation coefficient > 0.5. Finally, a tumour cell MOMP signature, MOMP.Sig, consisting of genes highly expressed in tumour cells and exhibiting a strong correlation with tumour cell MOMP activity, was developed (Figure 4A,B and Table S9).

### 2.7 Development of machine learning models

Principal component analysis (PCA) was initially performed on the entire set of bulk RNA‐Seq datasets from diverse ICI cohorts. The PCA plots representing each cohort were annotated with colour markers, vividly demonstrating the stratification of the immunotherapy cohorts into two distinct groups. The Combat method was then applied to these two groups to mitigate batch effects (Figure S4A,B).^52^ The first group consisted of a pan‐cancer cohort encompassing six tumour types (GBM, NSCLC, RCC, SKCM, STAD and UC) from 13 immunotherapy cohorts. Subsequently, this group was randomly divided into training (*n* = 710; 70%) and test (*n* = 305; 30%) sets for model development and evaluation purposes. The second group comprised two tumour types (BRCA and RCC) from two independent immunotherapy cohorts (*n* = 241), which were specifically designated for additional validation of the developed models (Figure 4C). To validate the predictive value of MOMP.Sig in the immunotherapy response, various classification machine learning models from Scikit‐Learn were employed. The selected models included logistic regression, stochastic gradient descent classifier, support vector classification, k‐nearest neighbours classification, radius‐based neighbours classification, decision tree classifier, gradient boosting classifier, histogram‐based boosting classifier (HistGradientBoost), random forest classifier, nu‐support vector classification, extremely randomised trees classifier, multilayer perceptron classifier and Gaussian process classification (Table S5). Each model underwent ninefold cross‐validation on the training cohort and was subsequently evaluated on the test cohort. Model performance was assessed based on the area under the curve (AUC) in both the training and test cohorts, and the optimal model was chosen. Another validation cohort was included to validate the best model's predictions.

### 2.8 MRs associated with ICI resistance

To elucidate the underlying mechanisms contributing to immunotherapeutic ineffectiveness, the immunotherapy response and resistance were investigated. Subsequently, the specific transcriptional regulators responsible for initiating transitions between these two states and maintaining the stability of the immune resistance state were investigated through their mechanistic regulation. The ARACNe‐AP algorithm was used to construct a training and test cohort gene‐regulatory network of transcription factors (TFs), cotranscription factors and signalling proteins. The two‐state‐specific TF activity of the responders versus nonresponders accurately predicted in the model was computed by using the VIPER algorithm. These validated analyses help to elucidate MR proteins whose abnormally elevated activities are critical for the maintenance of immunotherapy resistance.

### 2.9 Pan‐cancer CRISPR ICI datasets

To improve the efficacy of immunotherapy in nonresponders predicted by the model and to explore potential immune resistance mechanisms, CRISPR screening data related to immunotherapy were systematically collected. These datasets included four in vivo CRISPR screening datasets and one in vitro dataset that included breast cancer,^53^ lung cancer,^54^ melanoma,^55^ liver cancer^56^ and colorectal cancer samples.^57^ The analysis of these datasets aimed to identify crucial MRs that contribute to immune resistance.

### 2.10 Histology and immunohistochemistry

For immunohistochemistry (IHC), paraffin‐embedded sections from 79 patients with SKCM and 38 patients with triple‐negative breast cancer (TNBC) before immunotherapy were obtained from Sun Yat‐sen University Cancer Center (SYSUCC) (Tables S6 and S7). The use of all samples in this study was authorised by the Medical Ethics Committee of SYSUCC. The integrity of the tissues was confirmed through haematoxylin and eosin staining of the sections. These sections were then subjected to antigen retrieval by immersion in EDTA citrate buffer (pH 6.0 or pH 8.0) and microwave heating. Following antigen retrieval, the sections were incubated overnight at 4°C with an anti‐FOXO1 antibody (1:500 dilution, ET1608‐25; HuaAn Biotechnology, Hangzhou, China). A negative control using normal rabbit IgG was included for specificity validation. Subsequently, the sections were treated with an HRP‐conjugated secondary antibody for 30 min and visualised with diamino‐benzidine solution (ZSGB‐Bio). Nuclear counterstaining was performed with haematoxylin. To assess the FOXO1 expression level in patients, we initially employed the classifier function of the HALO platform for intelligent identification of tissue slices, accurately delineating tumour and non‐tumour regions.^58^ Subsequently, the CytoNuclear module was utilised to analyse the staining intensity and distribution range of FOXO1 in the nuclei of tumour cells, obtaining detailed histochemical scoring (*H*‐score) at the nuclear level.^58^ Finally, patients were grouped into high‐expression and low‐expression groups according to the mean *H*‐score of FOXO1.

### 2.11 Quantitative real‐time PCR

Quantitative PCR (qPCR) was used to assess the expression of MOMP‐related genes in different types of tumour cell lines after 48 h of FOXO1 inhibitor treatment (T6762; TargetMol Chemicals, USA). Initially, total RNA was extracted from the cells using an RNA Purification Kit (B0004D; EZBioscience). The extracted RNA was then reverse transcribed into cDNA using a HiScript II Q RT Kit (R223‐01; Vazyme Biotech, China). Subsequently, qPCR was performed using ChamQ SYBR qPCR Green Master Mix (Q311‐03; Vazyme Biotech) and various target gene primers and primers for the housekeeping gene GAPDH. The reactions were performed on a Roche LightCycler 480, and the mRNA levels of the target genes were normalised relative to the GAPDH level. All qPCRs were carried out in triplicate. All the primers used for qPCR are listed in Table S8.

### 2.12 Statistical analysis

Spearman correlation was used to evaluate the relationships between average MOMP activity in diverse cancer types and IPS, immune effector cells, immune suppressor cells and immune‐related molecules, and Pearson correlation was utilised for all other analyses investigating the relationships between two variables. Univariate and multivariate Cox regression analyses were employed to estimate the hazard ratio and 95% confidence interval for MOMP activity with respect to survival. Additionally, the odds ratio for MOMP activity concerning the response to immunotherapy was estimated using the R software package ‘glmnet’. with the family parameter set to binomial. For continuous variables, the Wilcoxon rank‐sum test and Kruskal‒Wallis test were utilised to estimate differences between two groups and among multiple groups, respectively. For categorical variables, either the chi‐squared test or Fisher's exact test was employed to examine differences. Survival probability was compared using Kaplan‒Meier curves and log‐rank tests. All the statistical analyses were performed using R version 4.2.3. The significance level for p values was set at *p* < .05, indicating statistical significance in all two‐sided tests.

## 3 RESULTS

### 3.1 MOMP activity varies across cancers and predicts patient survival

To quantify MOMP activity in tumour samples, we established specific pathways reflecting MOMP activity (Figure 1A and Table S1), including apoptosis, pyroptosis, cGAS/STING, inflammasome and NF‐kB pathways, through previous literature mining and summarising.^8^, ^59^, ^60^ Using 10 118 samples from 30 solid cancer types in TCGA and applying the ssMWW–GST algorithm,^24^ we systematically deconstructed the pan‐cancer MOMP activity profiles.

By applying our methods, we observed substantial variation in MOMP activity across different cancer types (Figure 1B). Subsequently, we performed an additional assessment of the clinical significance of MOMP activity. Univariate and multivariate Cox analyses were employed to investigate the association between MOMP activity and survival prognosis across different tumour types. The results demonstrated a notable positive impact of MOMP activity on prognosis, where higher MOMP activity was associated with lower hazard ratios for overall survival (OS), disease‐specific survival and progression‐free interval (PFI) (Figures 1C and S1). Pan‐cancer patients were stratified into two groups based on the mean MOMP activity. Patients in the high‐MOMP activity group had longer OS, PFI and disease‐free interval compared with those in the low‐MOMP activity group (Figure 1D). Thus, MOMP activity exhibits significant potential for predicting survival prognosis.

### 3.2 MOMP activity is positively associated with anti‐tumour immunity

**FIGURE 2:**
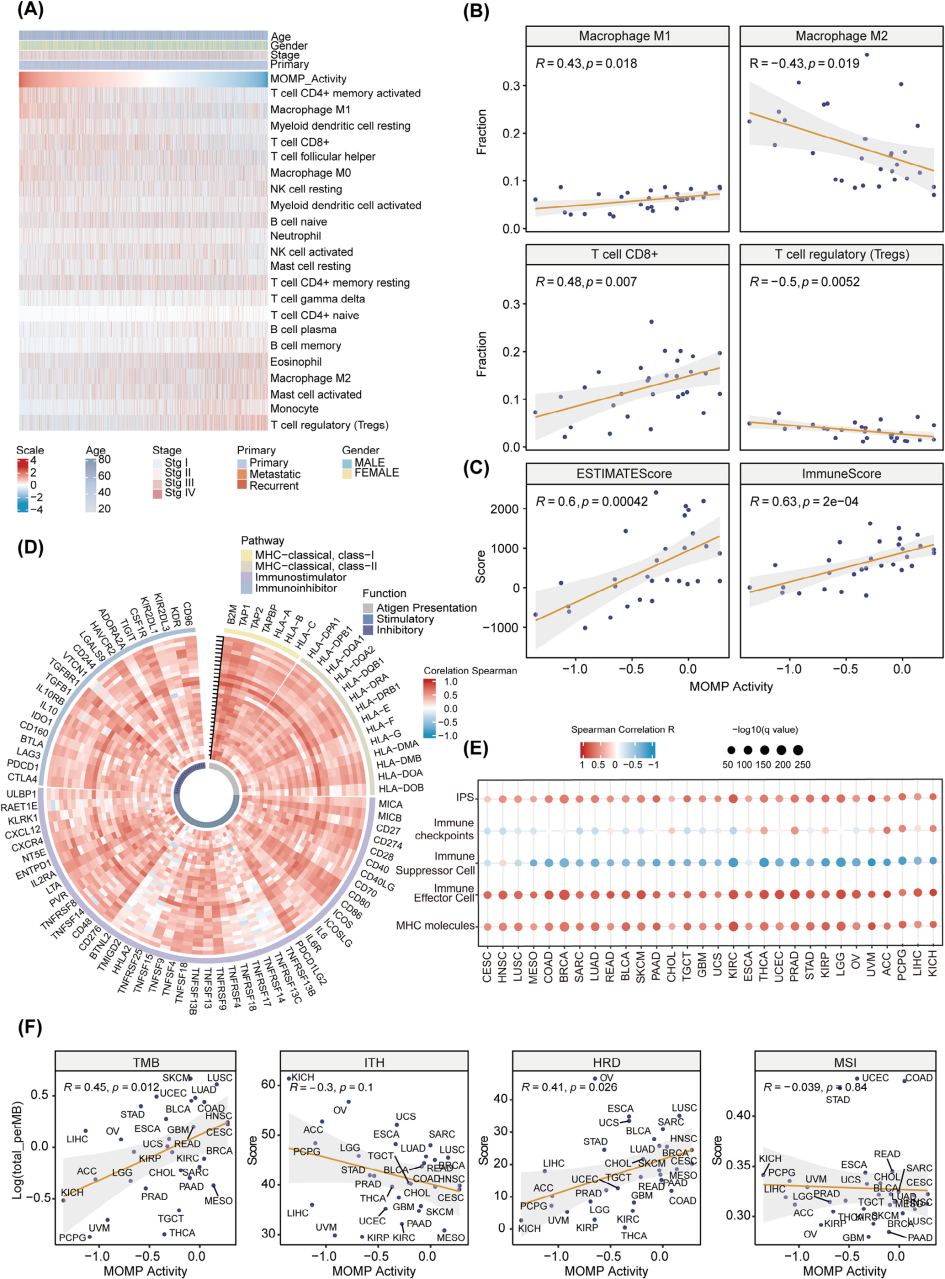
MOMP activity is positively associated with anti‐tumour immunity. (A) The component profiles of the tumour immune microenvironment from the CIBERSORT algorithm in the pan‐cancer cohort. (B) Linear regression analysis depicting correlations between the fractions of M1 macrophages, M2 macrophages, CD8+ T cells and Tregs, as quantified by the CIBERSORT algorithm, and MOMP activity across a pan‐cancer cohort. (C) Linear regression analysis depicting the correlation between the ESTIMATE score and immune score, as calculated by the ESTIMATE algorithm, and MOMP activity across a pan‐cancer cohort. (D) Association between immune‐related genes and MOMP activity across 30 solid cancer types. The inner to outer axes marked with black arrows in the Circos plot represent distinct cancer types, with corresponding cancer types labelled on the *x*‐axis of plot E. (E) The correlation between MOMP activity in 30 solid cancer types and the IPS obtained from TCIA, along with the infiltration status of immune‐related cells. (F) The correlation between average MOMP activity and the average TMB, HRD score, ITH and MSI across 30 solid cancer types. HRD, homologous recombination deficiency; IPS, immune phenotype score; ITH, intra‐tumour heterogeneity; MOMP, mitochondrial outer membrane permeabilisation; MSI, microsatellite instability; TCIA, The Cancer Immunome Atlas; TMB, tumour mutational burden.

Considering that the induction of MOMP in tumour cells initiates cell death and immune activation and given the robust correlation between MOMP activity and enhanced prognosis in the pan‐cancer cohort, we hypothesised that MOMP incidence contributes to the establishment of an anti‐tumour TIME. To investigate the specific correlation between MOMP activity and the TIME, we employed the CIBERSORT algorithm to profile infiltrating immune cells in cancer patients within the pan‐cancer cohort (Figure 2A). Subsequently, we calculated the correlation between MOMP activity and different immune cell infiltration fractions (Figure S2A). We identified a positive correlation between MOMP activity and immune cells, including M1 macrophages and CD8+ T cells, all of which are intricately linked to anti‐tumour immunity. In contrast, a negative correlation was observed between M2 macrophages and Tregs, which are associated with immune suppression (Figure 2B). Within the TIME, heightened MOMP activity corresponds to increased immune scores and ESTIMATE scores (Figure 2C).

Due to the inherent connection between tumour purity and immune cell infiltration,^61^ we divided patients into high and low groups based on average tumour purity, and the results indicated that MOMP activity was significantly positively correlated with the immune cell infiltration fraction (Figure S2B). This finding underscores the pivotal role of MOMP activity in shaping tumour immunity. To further deepen our understanding of the impact of MOMP activity on the TIME, we conducted an analysis using TCIA to examine the expression levels of immune‐related molecules, the infiltration pattern of immune cells and the IPS.^34^ We observed a noteworthy positive correlation between MOMP activity and the expression of antigen presentation‐related molecules and immune stimulation‐related molecules (Figure 2D). Additionally, we also found that increased MOMP activity was associated with increased infiltration of immune effector cells and reduced levels of immunosuppressive cells (Figure 2E). Moreover, following the comprehensive evaluation of the impact of immune‐related molecules and immune cells, the IPS, which is indicative of the promotion of anti‐tumour immune responses, exhibited a positive correlation with MOMP activity (Figure 2E). These results indicate that the increase in MOMP activity contributes to the formation of an anti‐tumour immune environment. High MOMP activity not only predicts a favourable prognosis but also suggests potential benefits from immunotherapy.

Finally, we analysed the impact of tumour multi‐omics features on MOMP activity. Interestingly, we observed a positive correlation between TMB and HRD and between TMB and MOMP activity, whereas ITH and MSI exhibited no apparent association with MOMP activity (Figure 2F). Currently, there is a lack of comprehensive explanations for these correlations, and their underlying mechanisms remain to be further investigated.

### 3.3 MOMP activity is positively associated with immunotherapy response

**FIGURE 3:**
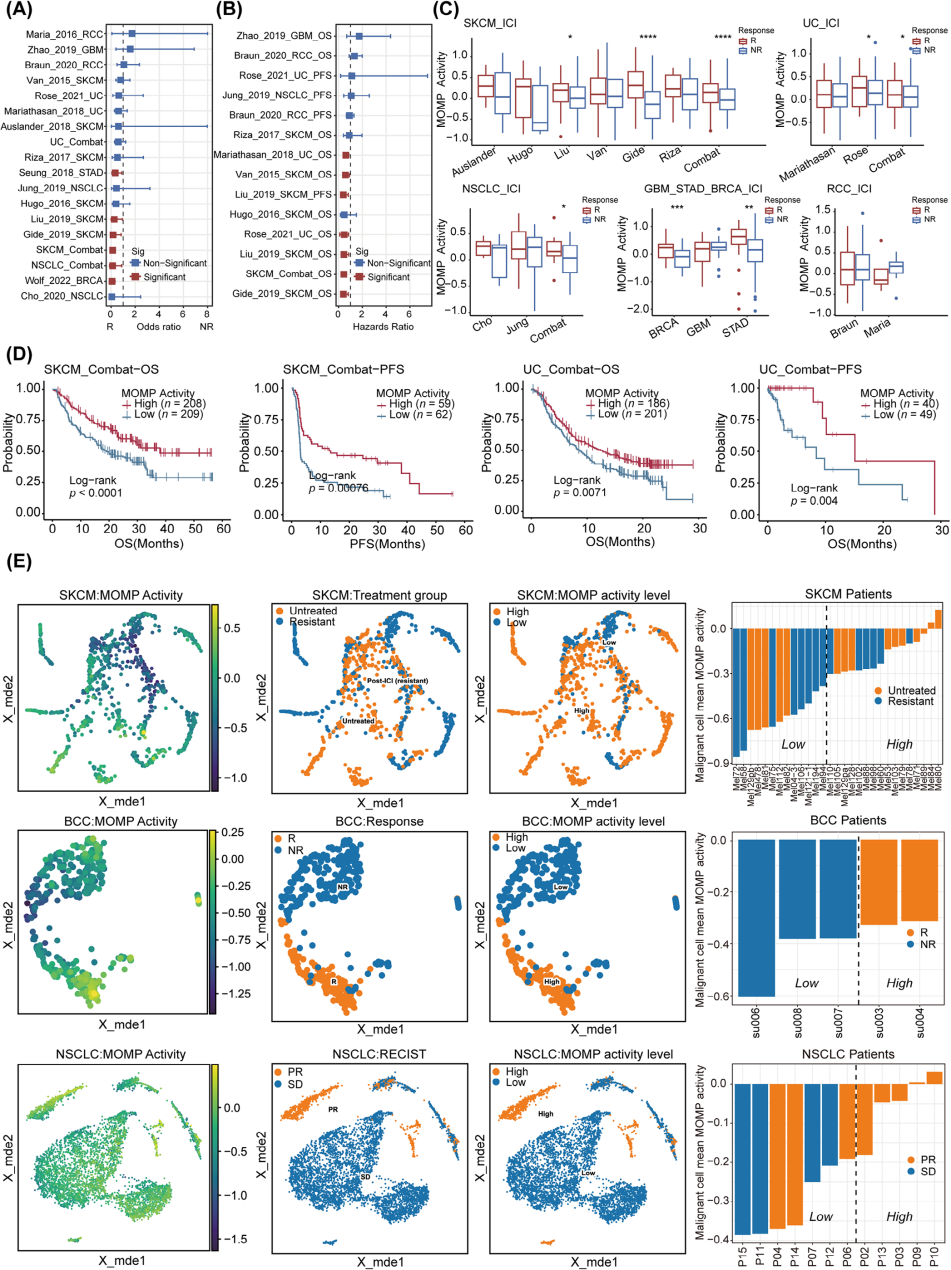
MOMP activity promotes the ICI response. (A) The effect size of the correlation with ICI response across various ICI cohorts for MOMP activity. (B) The hazard ratio for the association of MOMP activity with survival across ICI cohorts. (C) The MOMP activity of various ICI response phenotypes stratified by cancer type. The centre of the box plot represents the median, and the box boundaries indicate the 25th and 75th quantiles. Statistical significance was evaluated by a two‐sided Wilcoxon rank sum test. (D) Kaplan–Meier curve of patient survival stratified by the average MOMP activity for the SKCM and UC ICI cohorts. (E) Dimension reduction plots demonstrating the distribution of MOMP activity in tumour cells in the SKCM, BCC and NSCLC ICI scRNA‐Seq datasets (left). The dimension reduction plots label tumour cells according to the type of immunotherapy response (centre). The dimension reduction plots label tumour cells according to the level of tumour cell MOMP activity (right). The bar chart presents the distribution of average MOMP activity in the tumour cells of patients and their response to immunotherapy. *p < .05, **p < .01; *** *p* < .001; ****, *p* < .0001. BCC, basal cell carcinoma; ICI, immune checkpoint inhibitor; MOMP, mitochondrial outer membrane permeabilisation; NR, nonresponse; NSCLC, non‐small cell lung cancer; PR, partial response; R, response; SD, stable disease; SKCM, skin cutaneous melanoma; UC, urothelial carcinoma.

Increased MOMP activity promotes an anti‐tumour TIME, thereby contributing to an enhancement in the response to tumour immunotherapy. To systematically investigate the impact of MOMP activity on tumour immunotherapy efficacy, we assessed MOMP activity status, immunotherapy response and prognosis in a pan‐cancer ICI cohort (including patients with BRCA, GBM, NSCLC, RCC, SKCM, STAD and UC) (Table S2). Initially, we calculated the effect size of the correlation between MOMP activity and ICI response in each cohort. In the majority of the ICI cohorts (BRCA, NSCLC, STAD, SKCM), we observed that MOMP activity positively influenced the immunotherapy response (Figure 3A). Subsequently, based on the immunotherapy response of the patients, we classified them into responder and non‐responder groups. Notably, the average MOMP activity was greater in the responder group in the SKCM, UC, NSCLC, STAD and BRCA cohorts (Figure 3C). Next, a Cox proportional hazards model was used to assess the impact of MOMP activity on the prognosis of patients receiving immunotherapy. In the SKCM and UC cohorts, increased MOMP activity was linked to a reduced risk of mortality (Figure 3B). Importantly, after stratifying patients based on whether MOMP activity was greater than the mean value, we observed a superior survival prognosis in the high‐MOMP‐activity group within SKCM and UC (Figure 3D).

Unlike bulk RNA‐seq, which can only capture the average genetic profile of a diverse array of cells, scRNA‐seq technology provides transcriptomic information for each cell in a sample. This enables a comprehensive assessment of the relationship between MOMP activity and different cell types during tumour immunotherapy. First, the cell types across three independent scRNA‐Seq ICI cohorts (SKCM, BCC, NSCLC) were thoroughly annotated, and the MOMP activity of each cell was meticulously quantified (Figure S3A). We analysed the scRNA‐Seq ICI dataset from SKCM derived from pre‐immunotherapy biopsy samples. The dataset comprised 29 patients, including 15 who were not treated and 14 who exhibited ICI resistance. Despite the absence of immunotherapy responders, considering the potential presence of ICI responders in the untreated group, we conducted comparative examinations. The results revealed a greater incidence of tumour cells with lower MOMP activity in the ICI‐resistant cohort, whereas tumour cells with higher MOMP activity were more abundant in the untreated cohort (Figures 3E and S3B). This phenomenon was not observed in immune cells or stromal cells (Figure S3B). Patients were divided into high and low MOMP activity groups according to the average MOMP activity of the tumour cells. Among the 16 patients with high‐level MOMP activity, 10 were untreated and six were immunotherapy‐resistant. In contrast, among the 13 patients with low‐level MOMP activity, five were untreated and eight were immunotherapy tolerant (Figure 3E). This finding suggested a potential correlation between lower MOMP activity in tumour cells and ICI resistance. In the scRNA‐Seq dataset from pre‐immunotherapy biopsy samples of BCC patients, tumour cells from two ICI responders exhibited significantly greater average MOMP activity than did those from three ICI non‐responders, with no significant difference observed in non‐tumour cells (Figure S3B). Moreover, consistent conclusions were drawn even in the scRNA‐Seq cohort after immunotherapy. The average MOMP activity of tumour cells from eight ICI responders in the PR subgroup was greater than that of tumour cells from four ICI non‐responders in the SD subgroup (Figures 3E and S3B). A more pronounced discrepancy in MOMP activity levels was evident between the PR and SD subgroups. Similarly, based on the average MOMP activity of tumour cells, patients were divided into high‐ and low‐MOMP‐activity groups. Among the five patients with high‐level MOMP activity, all exhibited an immunotherapy response, whereas among the seven patients with low‐level MOMP activity, only three exhibited an immunotherapy response (Figure 3E). In conclusion, the use of scRNA‐Seq technology further confirmed the close association between tumour cell MOMP activity and the response to immune therapy.

### 3.4 Constructing MOMP.Sig using pan‐cancer scRNA‐Seq

**FIGURE 4:**
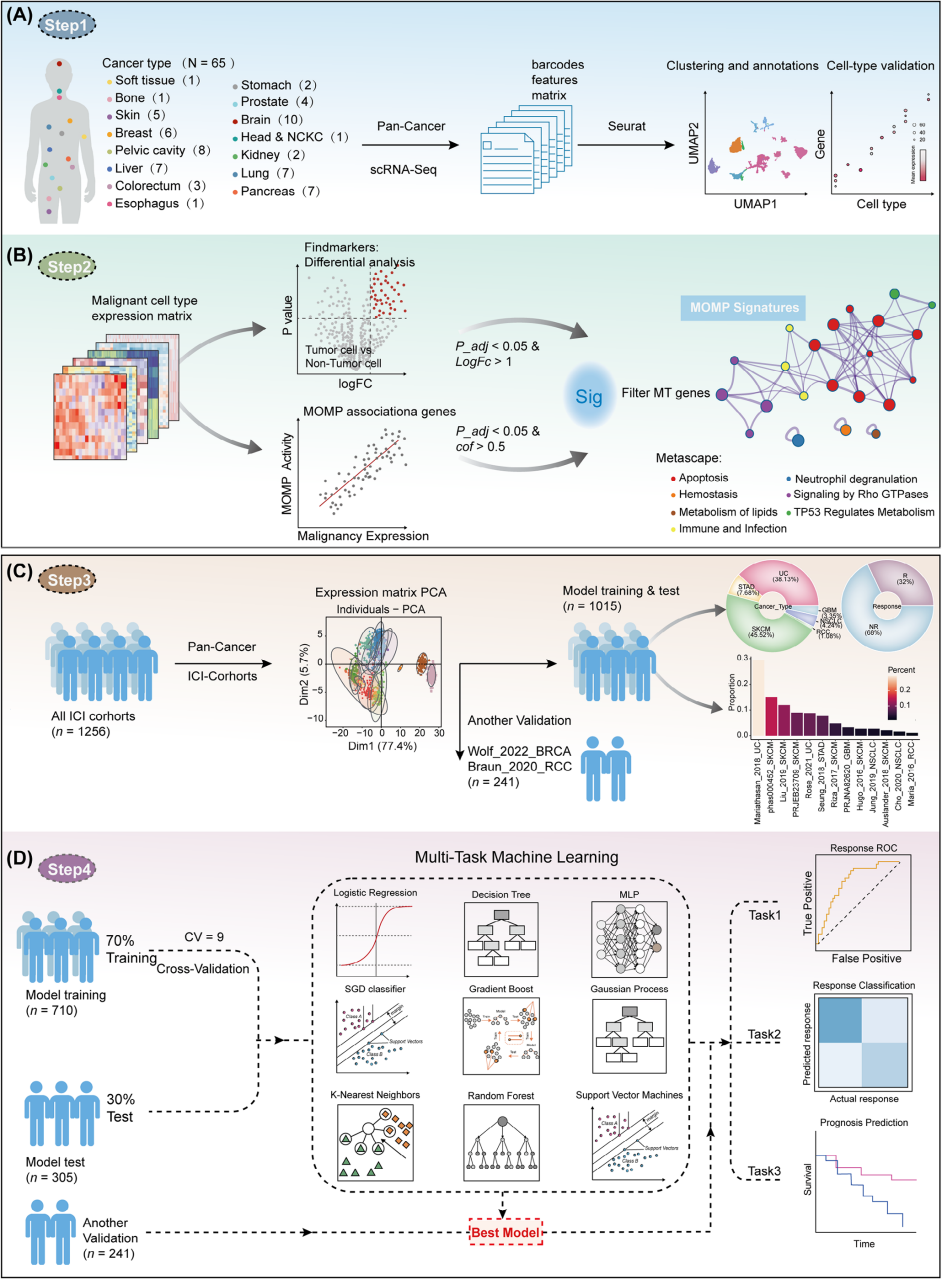
Development of MOMP.Sig and machine learning models. (A) A pan‐cancer scRNA‐Seq dataset used for obtaining the tumour MOMP activity signature (MOMP.Sig). (B) Flowchart for the development of MOMP.Sig using pan‐cancer scRNA‐Seq. (C) The integration process of the pan‐cancer ICI cohort for model training, testing and validation. (D) Overview of the process of developing machine learning models for predicting ICI response and survival. ICI, immune checkpoint inhibitor. NR, nonresponse; R, response.

The scRNA‐seq ICI cohort confirmed a significant correlation between tumour cell MOMP activity and response to immunotherapy. To accurately determine tumour cell MOMP activity from bulk RNA‐Seq data and minimise non‐tumour cell interference, it is crucial to develop a specific signature for tumour cell MOMP activity. Pan‐cancer scRNA‐Seq data, encompassing 26 solid cancer types originating from 15 distinct anatomical locations, offer valuable insights into constructing tumour cell MOMP activity signatures (Figure 4A and Table S4). Utilising the FindAllMarkers function in Seurat, we analysed highly expressed feature genes specifically in tumour cells (Figure 4B). Subsequently, genes closely associated with MOMP activity in tumour cells were extracted from these feature genes. The genes that are specifically expressed in tumour cells and strongly linked to tumour cell MOMP activity constitute the MOMP activity signature, MOMP.Sig (Figure 4B and Table S9). MOMP.Sig is anticipated to provide a more accurate representation of MOMP activity in tumour cells. To validate the relevance and accuracy of MOMP.Sig in MOMP activity, we investigated the biological functions associated with MOMP.Sig by Metascape.^62^ We found that it is primarily associated with apoptosis, metabolism and immune functions, closely resembling the biological functions influenced by MOMP (Figure 4B). This finding substantiates the accuracy and reliability of MOMP.Sig.

### 3.5 Building machine learning models based on MOMP.Sig

Given that MOMP.Sig more accurately reflects tumour cell MOMP activity, we investigated its clinical value in predicting immunotherapy response within the pan‐cancer ICI cohort. All 15 cohorts were divided into three datasets: a training set (*n* = 710), a test set (*n* = 305) and another validation set (*n* = 241). The analysis process flowchart is depicted in Figure 4C,D. The training and test sets included a pan‐cancer cohort of six tumour types (GBM, NSCLC, RCC, SKCM, STAD and UC) from 13 immunotherapy cohorts utilised for model development and evaluation purposes. Another validation set comprised two tumour types (BRCA and RCC) from two independent immunotherapy cohorts, which were specifically designated for further validation of the developed models. Subsequently, using the expression profiles of MOMP.Sig from the immunotherapy cohort, we developed multiple machine learning‐based models to predict both the immunotherapy response and patient prognosis. Specifically, the training set underwent ninefold cross‐validation for model training, whereas the test set was used for model testing. Another validation set was used for independent validation of the model.

**FIGURE 5:**
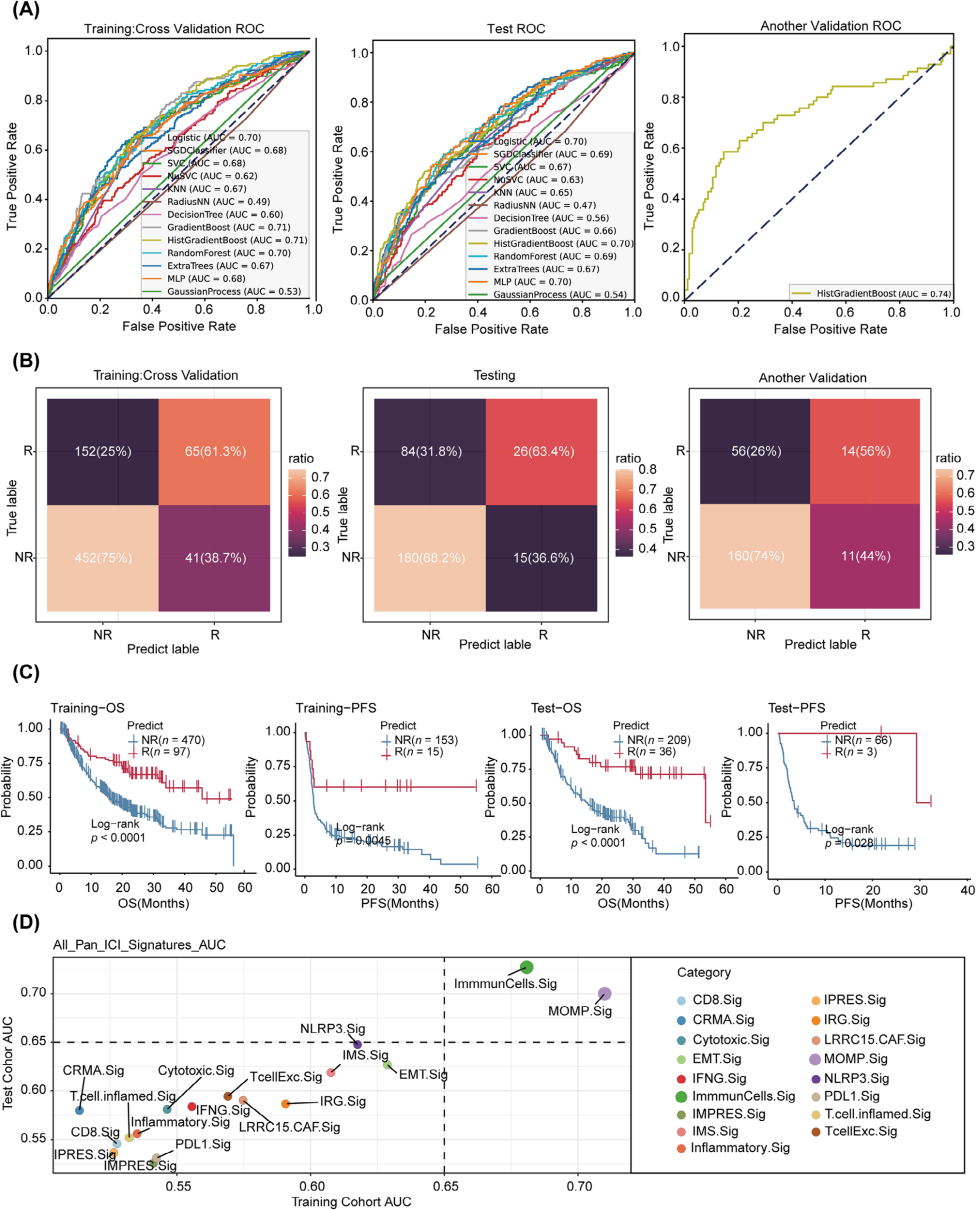
Prediction results of ICI response and survival using the machine learning model based on MOMP.Sig. (A) The ROC curves demonstrating the predictive performance of the machine learning model in the training, testing and validation cohorts. (B) Confusion matrices of the training, testing and validation cohorts based on the HistGradientBoost machine learning model. (C) Kaplan‒Meier analyses of OS and PFS according to the model‐predicted ICI response in the training and test cohorts. (D) Comparison of the immunotherapy predictive performance of MOMP.Sig with other pan‐cancer ICI response predictive signatures in the training and test cohorts. ICI, immune checkpoint inhibitor; NR, nonresponse; OS, overall survival; PFS, progression‐free survival; R, response; ROC, receiver operating characteristic.

The results demonstrated that among the various machine learning models, the HistGradientBoost model was the optimal model, exhibiting consistently high discriminative power on both the training and test sets, with AUC values of 0.71 and 0.70, respectively. Further validation on another validation set confirmed the model's predictive ability, with an AUC of 0.74 (Figure 5A). The confusion matrix consistently illustrated that the HistGradientBoost model accurately predicted immunotherapy response categories across the training, test and additional validation sets (Figure 5B). Further assessment was conducted to explore the relationships between the predicted immunotherapy response categories and patient prognosis. Survival analysis revealed that in the training and test sets, patients who were predicted to be non‐responders had poorer OS and progression‐free survival (PFS) than patients who were predicted to be responders (Figure 5C). In another validation set, patients who were predicted to be non‐responders also had worse PFS than patients who were predicted to be responders (Figure S4C). Therefore, utilising MOMP.Sig enables the prediction of immunotherapy outcomes. To further assess its predictive efficacy, we compared MOMP.Sig with 16 other previously reported immunotherapy response signatures (Tables S9 and S10).^5^, ^35^, ^37^, ^63^, ^64^, ^65^, ^66^, ^67^, ^68^, ^69^, ^70^, ^71^, ^72^, ^73^ The results indicated that within the pan‐cancer ICI cohort, only MOMP.Sig and ImmunCells.Sig exhibited good AUC values in both the training and test cohorts, with other signatures falling below 0.65 in AUC (Figure 5D). Furthermore, in comparison with ImmunCells.Sig, MOMP.Sig achieved an AUC of 0.7 in both the training and test cohorts, while the AUC of ImmunCells.Sig was less than 0.7 in the training cohort (Figure 5D). In summary, the findings suggest that among these signatures for predicting immunotherapy response, MOMP.Sig stands out as the optimal signature. This underscores MOMP.Sig is an effective biomarker capable of accurately predicting the clinical outcomes of ICIs based on pretreatment tumour samples.

### 3.6 The identification of immune resistance targets

**FIGURE 6:**
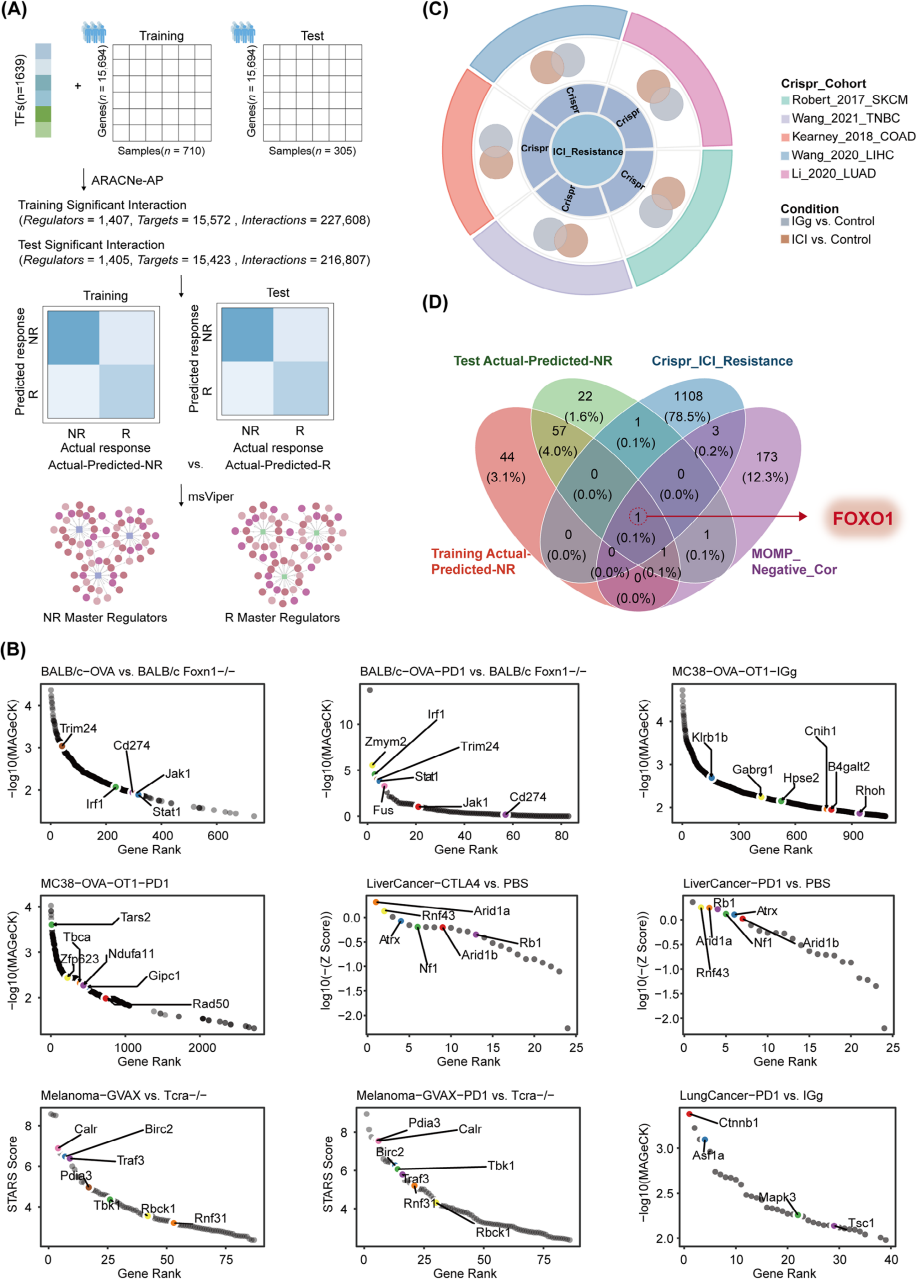
Integration of pan‐cancer ICI CRISPR screening data and MOMP.Sig modelling to explore key MRs involved in immune resistance. (A) Schematic diagram and workflow for identifying MRs associated with immune resistance using the ARACNe‐AP and msVIPER methods. (B) Abundance distribution of sgRNAs in ICI CRISPR screens for breast cancer, colon cancer, liver cancer, melanoma and lung cancer. (C) Integration of immune resistance genes from pan‐cancer ICI CRISPR data. (D) Analysis of the overlap between immune resistance MRs, genes negatively correlated with tumour cell MOMP activity, and immune resistance genes revealed by a pan‐cancer ICI CRISPR screen. ICI, immune checkpoint inhibitor; MR, master regulator; NR, nonresponse; R, response.

The HistGradientBoost model based on MOMP.Sig, it is anticipated that a notable subset of patients who are predicted to respond to immunotherapy will attain substantial clinical efficacy. Conversely, a substantial proportion of patients predicted to be nonresponsive to immunotherapy are expected to derive minimal direct benefits from interventions in immunotherapeutic approaches. This underscores the critical need to elucidate the fundamental mechanisms underpinning immunotherapeutic ineffectiveness, thereby fostering strategies to expand the clinical benefit of immunotherapy. To identify the transcriptional machinery driving immunotherapy resistance, we utilised ARACNe‐AP and VIPER (Figure 6A). In the training cohort, we identified 103 pivotal MRs associated with promoting immune resistance, while 83 key MRs were confirmed in the test cohort, 59 of which were consistently observed in both cohorts (Figure S4D,E and Table S11).

**FIGURE 7:**
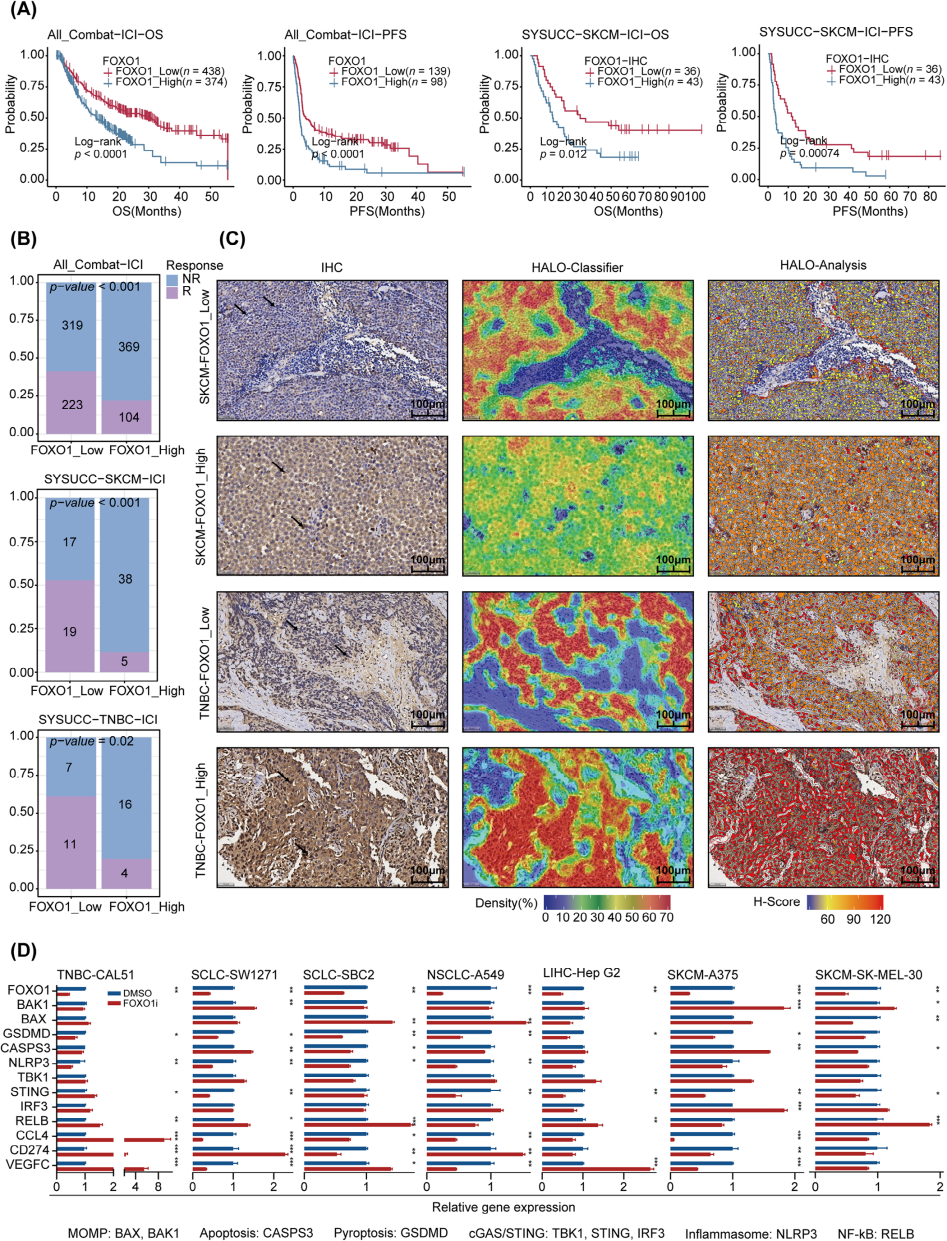
Clinical cohort validation of the impact of FOXO1 on immunotherapy efficacy. (A) Kaplan–Meyer analysis of OS and PFS in the pan‐cancer ICI cohort and the SYSUCC‐SKCM ICI cohort according to FOXO1 expression level. (B) ICI responses based on the expression levels of FOXO1 in the pan‐cancer ICI cohort and the SYSUCC‐SKCM and TNBC ICI cohorts. (C) IHC analysis of FOXO1 in SYSUCC‐SKCM and TNBC pathology sections in the HALO platform. Density refers to the density of tumour cells in tumour tissue. The *H*‐score was used to quantify the level and distribution of FOXO1 within the nuclei of tumour cells. Scale bar, 100 µm. (D) Changes in the mRNA levels of MOMP and immune‐related molecules in different tumour cell lines (TNBC, SCLC, NSCLC, LIHC and SKCM) following FOXO1 inhibitor treatment. The data are presented as the mean ± SD. Statistical significance was evaluated by an unpaired Student's *t*‐test. *p < .05; **p < .01; ***p < .001. FOXO1i, FOXO1 inhibitor; ICI, immune checkpoint inhibitor; IHC, immunohistochemistry; LIHC, liver hepatocellular carcinoma; NR, nonresponse; NSCLC, non‐small cell lung cancer; OS, overall survival; PFS, progression‐free survival; R, response; SCLC, small cell lung cancer; SKCM, skin cutaneous melanoma; TNBC, triple‐negative breast cancer.

To select the most discriminating MRs among these candidate MRs, we initially extracted genes from the comprehensive pan‐cancer scRNA‐Seq dataset where the correlation coefficient between tumour cell gene expression levels and MOMP activity was significantly less than −0.1 and that appeared in at least five scRNA‐Seq datasets (Table S11). These genes possess the potential to inhibit tumour cell MOMP activity, thereby suppressing immune activation. Subsequently, we systemically collected CRISPR immunotherapy datasets from diverse cancer types, including breast cancer,^53^ liver cancer,^56^ lung cancer,^54^ melanoma^55^ and colon cancer.^57^ By analysing the sgRNA abundance in the CRISPR screening results from the control and immunotherapy groups, we identified key genes that promote immune resistance (Figure 6B,C). Integrating candidate MRs, genes negatively correlated with tumour cell MOMP activity, and CRISPR screening, FOXO1 emerged as the most significant MR associated with the maintenance of immunotherapy resistance (Figure 6D). In the pan‐cancer ICI cohort, patients were stratified into FOXO1 high‐ and low‐expression groups based on the average level of FOXO1 expression. Within the high‐expression group, there was a significantly greater number of patients exhibiting immunotherapy resistance than in the low‐expression group (Figure 7B). Additionally, survival analysis revealed that the high‐expression group exhibited significantly reduced OS and PFS compared with the low‐expression group (Figure 7A).

### 3.7 Clinical validation of the role of FOXO1 in immunotherapy

To further validate the impact of FOXO1 expression on immunotherapy efficacy from a clinical perspective, we collected pre‐immunotherapy pathological tissue slices from 79 SKCM patients and pathological biopsy slices from 38 TNBC patients at SYSUCC (Tables S6 and S7). FOXO1 IHC was performed, and immunohistochemical images, as well as FOXO1 expression levels, were assessed using the HALO platform (Figure 7C). Patients were subsequently categorised into FOXO1 high‐expression and low‐expression groups based on the mean *H*‐score. Consistent with previous analyses, a greater proportion of immunotherapy‐resistant patients were in the FOXO1 high‐expression group in both the SKCM and TNBC cohorts (Figure 7B). Additionally, in the SKCM cohort, patients in the FOXO1 high‐expression group had poorer prognoses in terms of OS and PFS (Figure 7A). In the pan‐cancer cohort, the AUC of the ROC curve for predicting immunotherapy response based on FOXO1 expression levels was 0.66 (Figure S5A). In SYSUCC‐SKCM and TNBC patients, the predicted ROC curve AUC of immunotherapy response based on the IHC *H*‐score were 0.76 and 0.90, respectively (Figure S5B,C).

The inhibition of FOXO1, a critical molecule in immune resistance, can induce immune activation or promote tumour cell death, thereby enhancing the efficacy of immunotherapy. In cellular experiments involving seven cell lines from five different tumours treated with a FOXO1 inhibitor (1 µM, 48 h), FOXO1 expression was variably suppressed. Key molecules associated with MOMP activity increased to varying degrees across different cell lines, and there were significant alterations in the expression of immune‐related molecules (except for SK‐MEL‐30) (Figure 7D). In CAL‐51 cells, the expression of STING, RELB, CCL4 and CD274 increased. SW1217 cells exhibited increased expression of BAK1, CASPS3, RELB and CD274. SBC exhibited increased levels of BAX and RELB. A375 cells exhibited increased expression of BAK1, CASPS3 and IRF3. SK‐MEL‐30 cells exhibited increased BAK1 and RELB expression. Hep‐G2 cells exhibited elevated RELB expression. Finally, the A549 cells exhibited increased expression of BAX and CD274.

These findings further substantiate the inhibitory effect of FOXO1 on anti‐tumour immunity and its regulatory effect on MOMP‐related molecules. Preliminary experiments indicate the need for thorough validation of the use of FOXO1 inhibitors in enhancing immunotherapy efficacy. Exploring the specific role and regulatory mechanisms of MOMP and immune‐related molecules will provide essential theoretical support for future complex experiments and clinical studies.

## 4 DISCUSSION

Although MOMP has been recognised as a pivotal step in both cell death and inflammation signalling within the mitochondrial network, its implications in the TIME and anti‐tumour immunity have not been systematically investigated. Our study revealed a positive correlation between tumour MOMP activity and anti‐tumour immunity, as well as the response to ICIs. Elevated MOMP activity is associated with increased expression of MHC‐related molecules, augmented infiltration of immune effector cells, reduced presence of immune suppressor cells and enhanced responsiveness to immunotherapy. Considering the influence of non‐tumour cells on tumour cell MOMP activity, scRNA‐seq in ICI cohorts reaffirms the favourable role of tumour cell MOMP activity. To precisely capture tumour cell MOMP activity from bulk RNA‐Seq data, we integrated pan‐cancer scRNA‐Seq data to formulate a robust MOMP activity signature that exhibited superior predictive accuracy in predicting ICI response compared with other investigated signatures. In addressing immunotherapy resistance, a comparative analysis of accurately predicted immunotherapy responders and non‐responders based on MOMP.Sig model, along with genes exhibiting a negative correlation with tumour cell MOMP activity from a comprehensive pan‐cancer scRNA‐Seq dataset and pan‐cancer immunotherapy CRISPR screening results, identified FOXO1 as a key MR involved in immune resistance. The inhibition of FOXO1, in conjunction with immunotherapy, holds promise for potential benefits in patients who are predicted to be nonresponsive to conventional therapy. In conclusion, our study not only underscores the pivotal role of the tumour MOMP in predicting tumour immunotherapy but also identifies potential key targets for overcoming immunotherapy resistance, offering valuable insights for refining immunotherapy strategies.

MOMP involves intricate mechanisms governed by precise temporal and spatial coordination and operates in a non‐equilibrium state.^74^ Currently, there is no standardised or precise experimental method for assessing its extent accurately.^18^, ^19^, ^20^, ^21^, ^22^ Studies indicate that MOMP occurs rapidly within cells, with a window of approximately 10 min according to cell biology research.^75^, ^76^ Additionally, varying degrees of MOMP result in diverse downstream effects, such as cell death and inflammatory responses.^60^ These factors collectively contribute to the challenges in accurately detecting MOMP. Moreover, existing examination methods primarily focus on live‐cell level detection, limiting their clinical oncology applications. In clinical settings, cells from excised tumour tissues rapidly undergo cell death after biopsy or surgery, complicating the accurate assessment of the original MOMP within the tumour tissue. Considering the pivotal function of MOMP in cellular demise and immune activation signalling networks, MOMP directly activates pathways involved in apoptosis, necroptosis, cGAS/STING, NF‐kB and inflammasome activation.^8^, ^59^, ^60^ In this study, we integrated these pathways using bioinformatics methods, building upon previous research approaches,^25^, ^26^, ^27^ to elucidate the level of MOMP within tumour tissues to better explore the relationship between MOMP in tumour tissues and tumour immunotherapy.

MOMP serves as a pivotal trigger for apoptosis, pyroptosis and proinflammatory signalling. Our comprehensive pan‐cancer analyses highlight the specific contribution of MOMP to shaping the landscape of the anti‐tumour TIME and enhancing the immunotherapy response. Tumour MOMP exhibits a unique anti‐tumour effect, perhaps stemming from the rapid and comprehensive nature of the process. Previous studies have proposed a detailed model explaining how MOMP initiates a caspase‐dependent positive feedback loop through caspase‐mediated BID protein cleavage, further promoting MOMP and ultimately resulting in comprehensive MOMP.^75^ It is noteworthy that the completion of MOMP typically takes only a brief 10 min.^75^, ^76^ High‐speed imaging of mitochondrial apoptosis suggests that MOMP may begin in discrete subpopulations of mitochondria and then rapidly propagate in a wave‐like manner, traversing all mitochondria throughout the entire cell.^77^, ^78^, ^79^ This complex process ultimately leads to irreversible cell death, concurrently releasing a substantial amount of tumour‐associated antigens, thereby activating anti‐tumour immunity. In contrast, apoptosis, pyroptosis and inflammatory signalling induced by chronic stress and sustained inflammatory stimuli eventually promote tumour progression and immune suppression.^80^, ^81^ Additionally, when glycolytic activity is enhanced, complete MOMP can be prevented (referred to as incomplete MOMP), partially compromising mitochondrial function, restoring energy metabolism levels, preventing metabolic collapse, promoting tumour cell survival and inhibiting the onset of tumour immunity.^82^ These research perspectives emphasise the critical role of comprehensive and rapid MOMP in enhancing anti‐tumour immune responses.

Additionally, TCGA pan‐cancer multi‐omics analysis revealed a positive correlation between MOMP activity and both TMB and HRD. However, direct research on the specific impact of TMB and HRD on mitochondrial membrane permeability is lacking. The relationships among these factors are intricate, and the underlying mechanisms remain largely unexplored. Only a limited number of studies have provided indirect evidence suggesting that TMB and HRD may influence mitochondrial function. TMB and HRD often exhibit a close correlation, with HRD frequently occurring alongside elevated TMB.^83^ Notably, homologous recombination repair (HRR) preserves mtDNA integrity and normal function, whereas mutations or defects in mtDNA can lead to dysfunction, impacting mitochondrial permeability and triggering MOMP.^84^ Furthermore, during mitochondrial fusion and fission processes, the segregation of wild‐type mtDNA from mutant mtDNA directly affects mitochondrial membrane permeability.^85^ These potential mechanisms may contribute to an increase in aberrant protein translation due to elevated HRD and TMB levels.^86^, ^87^ Upon entering mitochondria, aberrant proteins undergo posttranslational modifications such as oxidation, phosphorylation and ubiquitination,^88^ potentially inducing mitochondrial stress and exacerbating structural changes in MOMP. Moreover, other potential mechanisms may involve genes responsible for maintaining MOMP stability, such as BAX and BAK1.^89^ Mutations and HRD in molecules regulating these genes may enhance the function of BAX and BAK1, thereby promoting MOMP. In conclusion, additional research is necessary to comprehensively understand the intricate mechanisms by which the TMB and HRD impact the tumour MOMP.

The strong correlation between MOMP activity and anti‐tumour immunity underscores MOMP activity as a promising predictive marker for tumour immunotherapy. In this study, we introduce MOMP.Sig, a robust signature demonstrating effectiveness in predicting immunotherapy response and survival prognosis across the pan‐cancer immunotherapy cohort. Compared with various pan‐cancer signatures utilised in prior studies to predict the efficacy of tumour immunotherapies,^63^, ^64^, ^65^, ^66^, ^67^, ^68^, ^69^, ^70^, ^71^, ^72^, ^73^ MOMP.Sig exhibits excellent predictive accuracy. Unlike many previous pan‐cancer signatures derived from bulk RNA‐seq datasets, which are susceptible to interference from cellular components in the TIME and may compromise prediction accuracy, our approach integrates scRNA‐Seq datasets. This integration ensures a more precise reflection of tumour MOMP activity while minimising interference from non‐tumour cells in the TIME. An additional notable aspect is the utilisation of machine learning models^90^ for prediction in this study, which significantly enhanced the accuracy of predicting immunotherapy responses based on signature genes. This approach outperforms previous methods that rely on either the average expression of signature genes or a weighted assessment of signature genes.

The MOMP.Sig model not only aids in selecting suitable patients for immunotherapy but also plays a crucial role in shaping therapeutic strategies to overcome immune resistance. Using the ARACNe and Viper algorithms, we compared the activities of TFs accurately predicted in immunotherapy responders within the MOMP.Sig model with those in to identify immunotherapy‐resistant TFs. Integrating genes that exhibit a negative correlation with tumour cell MOMP activity and the pan‐cancer immunotherapy CRISPR screening results, FOXO1 emerged as the core TF involved in promoting immune resistance. Targeting FOXO1 to inhibit its expression has the potential to reverse immune resistance. Previous studies have characterised the function of FOXO1 as intricate and paradoxical. Primarily recognised as a tumour suppressor, FOXO1 exerts its tumour‐suppressive influence by regulating downstream PI3K/AKT signalling and attenuating the activities of the MYC and WNT signalling pathways.^91^ Paradoxically, FOXO1 may also promote tumourigenesis by directly upregulating the transcription of VEGFC, which is intricately linked with tumour progression and metastasis.^92^ Similarly, FOXO1 has a paradoxical impact on tumour immunity. In osteosarcoma, FOXO1 acts as an inhibitory molecule in the WNT signalling pathway, promoting the upregulation of CCL4 expression.^93^ This regulatory mechanism enhances the recognition of tumour cells by dendritic cells and subsequently activates CD8‐positive T cells, thereby strengthening anti‐tumour immunity.^94^ In gastric cancer, FOXO1 also suppresses VEGFA and other angiogenesis‐related molecules, inhibiting vascular formation in the tumour area and enhancing immune responses against tumour cells.^95^ These studies suggest that FOXO1 may play a positive role in immunotherapy. However, in colon and renal cancer cells, FOXO1 acts downstream of the PI3K/AKT signalling pathway, promoting the downregulation of MYC expression and inhibiting PD‐L1 expression.^91^, ^96^ Additionally, in prostate cancer cells, FOXO1 has been shown to promote the upregulation of VEGFC.^92^ Previous research has indicated that the downregulation of PD‐L1 and the upregulation of VEGFC hinder the effectiveness of immunotherapy.^97^, ^98^ This finding also emphasises the potentially important role of FOXO1 in immunotherapy resistance. Our clinical analysis of SKCM and TNBC ICI cohorts revealed a correlation between increased FOXO1 expression and immunotherapy resistance, suggesting the potential immunosuppressive role of FOXO1 in tumour immunotherapy. Furthermore, upon treating various types of tumour cell lines with FOXO1 inhibitors, we observed not only the upregulation of VEGFC, PD‐L1 and CCL4 but also varying degrees of increase in MOMP‐related molecules (such as BAX, BAK1, CASP3, STING, IRF3 and RELB). This finding suggested that in addition to its reported immune regulatory mechanisms, FOXO1 may have a potential regulatory role in MOMP. The immunosuppressive action of FOXO1 may also be achieved through the inhibition of MOMP. However, the exact mechanisms by which FOXO1 regulates MOMP and inhibits tumour immunotherapy require further detailed investigation.

However, several limitations of this study must be acknowledged and considered. First, in the pan‐cancer ICI cohort, the acquisition of comprehensive clinical data, including sex, age, tumour stage, TMB and ITH, was challenging. As a result, a multifactorial Cox proportional hazards analysis could not be performed to evaluate the influence of MOMP activity on survival and immunotherapy response. Second, within the scRNA‐Seq ICI cohort, BCC and SKCM cells underwent sequencing before immunotherapy, with only the BCC cohort encompassing tumour cells responsive and unresponsive to immunotherapy. The SKCM cohort included only tumour cells that were not treated with immunotherapy and those that were unresponsive to immunotherapy, whereas the NSCLC cohort included post‐immunotherapy sequencing data. Finally, the pan‐cancer ICI cohort in this study was limited to seven cancer types, with limited patient numbers for specific tumour types such as NSCLC, GBM and BRCA. These limitations constrain the predictive efficacy of MOMP.Sig for the immunotherapeutic outcomes. The predictive validity of MOMP.Sig requires additional confirmation through future prospective large‐scale ICI cohorts.

## 5 CONCLUSIONS

In summary, our comprehensive pan‐cancer analysis revealed the positive influence of tumour MOMP on anti‐tumour immunity. Through the integration of pan‐cancer scRNA‐Seq, we devised MOMP.Sig, a signature that more accurately represents the MOMP activity of tumour cells within the TIME. Compared with other pan‐cancer signatures, machine‐learning models demonstrated exceptional predictive efficacy for tumour immunotherapy. Finally, to address the critical challenge of immunotherapy resistance, we integrated MOMP.Sig‐based machine learning model with pan‐cancer CRISPR screening. This approach accurately identified a key MR, FOXO1, capable of promoting immune resistance. Analysis of clinical cohorts validated the close correlation of FOXO1 expression with immune resistance. Suppressing FOXO1 could potentiate the expression of MOMP‐related molecules, potentially fostering the development of anti‐tumour immunity. This lays the groundwork for precise immunotherapy.

## AUTHOR CONTRIBUTIONS

*Contributors Study concept and design*: Qingshan Chen, Jun Tang. *Acquisition of data*: Qingshan Chen, Junwan Wu, Jun Tang. *Statistical analysis*: Qingshan Chen, Fenglin Gao. *Interpretation of data*: Qingshan Chen, Fenglin Gao, Junwan Wu. *Drafting of the manuscript*: Qingshan Chen, Fenglin Gao. *Drafting of the figures*: Qingshan Chen, Fenglin Gao. *Critical revision of the manuscript*: Qingshan Chen, Fenglin Gao, Junwan Wu, Rong Deng, Jun Tang. *Technical support*: Kaiming Zhang, Tian Du, Yuhong Chen, Ruizhao Cai, Dechang Zhao. *Material support*: Rong Deng, Jun Tang. *Clinical data*: Qingshan Chen, Junwan Wu, Rong Deng, Jun Tang. *Study supervision*: Rong Deng, Jun Tang. *Data verification*: Rong Deng, Jun Tang. All authors read and approved the final version of the manuscript.

## CONFLICT OF INTEREST STATEMENT

The authors declare that there are no conflict of interests.

## ETHICS STATEMENT

The study protocol was approved by the Medical Ethics Committee of Sun Yat‐sen University Cancer Center (Medical Research Ethics Review No. G2021‐092‐01).

## Supporting information

Supporting information

Supporting information

## Data Availability

This project has not yet generated large‐scale experimental data. The analytical data utilised in this paper were downloaded from previous literature. The results of the analysis conducted in this study are provided in the supplementary tables. Detailed analysis and plotting codes for this research can be promptly requested from the author upon the publication of the article. Requests should be submitted via email to the author QSC.
