# Supplementary material for: Comprehensive pan‐cancer analysis of mitochondrial outer membrane permeabilisation activity reveals positive immunomodulation and assists in identifying potential therapeutic targets for immunotherapy resistance
Source: Clin Transl Med. 2024 Jun 20;14(6):e1735. doi: 10.1002/ctm2.1735 (PMC11187817; doi:10.1002/ctm2.1735)
Supplement: Supplementary file 1 — Supporting information [file CTM2-14-e1735-s001.docx]

**Supplementary Figures**

**This PDF file includes:**

FIGURES S1 to S4

[FIGURE S1 2](#_Toc162383037)

[FIGURE S2 3](#_Toc162383038)

[FIGURE S3 5](#_Toc162383039)

[FIGURE S4 6](#_Toc162383040)

[FIGURE S5 7](#_Toc162383041)

#
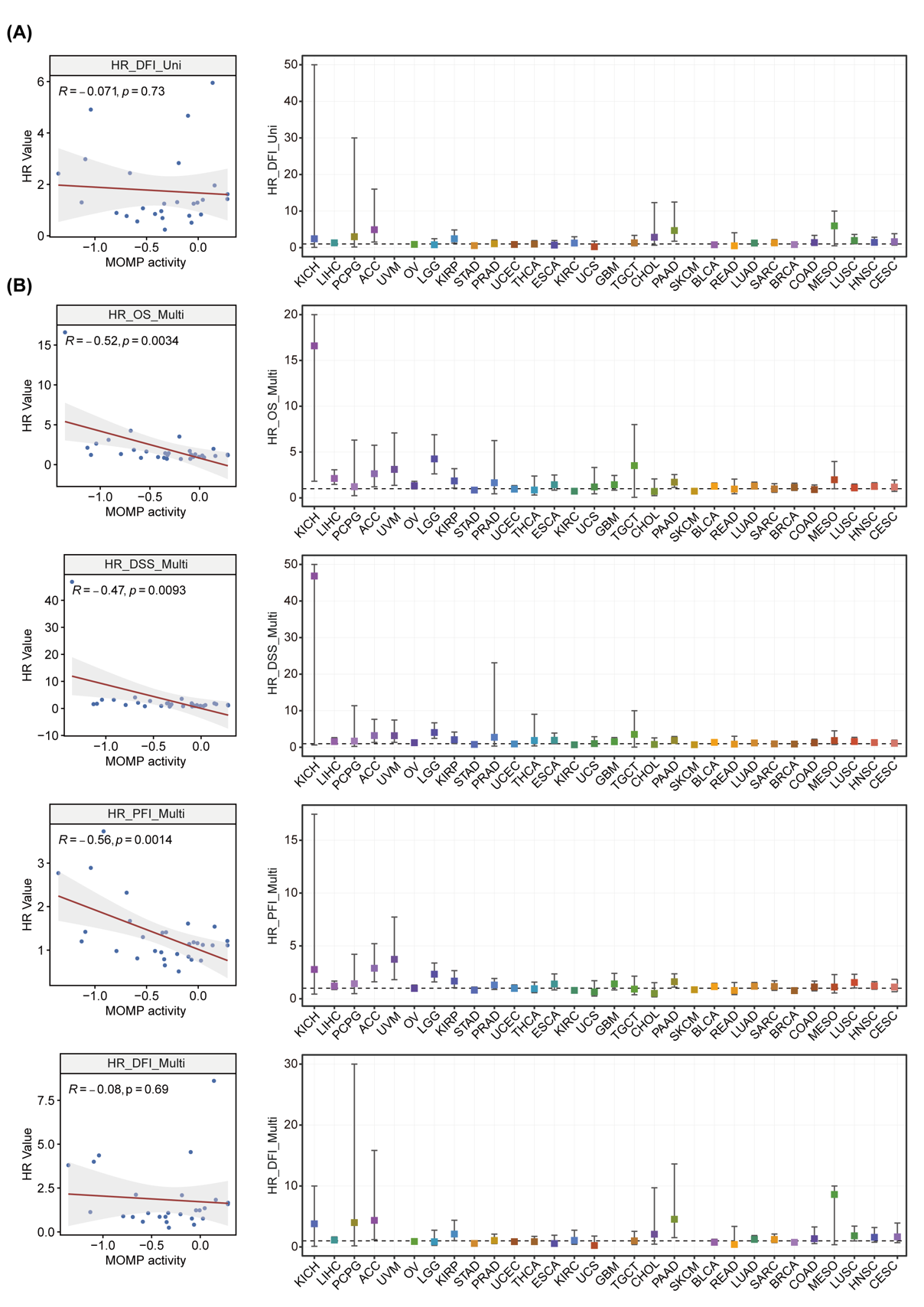
FIGURE S1

(A) Univariate Cox regression analysis elucidated the influence of tumor MOMP activity on the HR associated with the DFI, with cancer types ranked based on the average MOMP activity (right). Linear regression analysis quantified the relationship between the average MOMP activity in 30 solid cancer types from TCGA and their corresponding HR (±95% CI) for DFI (left). (B) Multivariable Cox regression analysis elucidated the influence of tumor MOMP activity on the HR associated with the survival, with cancer types ranked based on the average MOMP activity (right). Linear regression analysis quantified the relationship between the average MOMP activity in 30 solid cancer types from TCGA and their corresponding HR (±95% CI) for survival (left). MOMP, mitochondrial outer membrane permeabilization; TCGA, The Cancer Genome Atlas; OS, overall survival; DSS, disease-specific survival; DFI, disease-free interval; PFI, progression-free interval.

# FIGURE S2

**
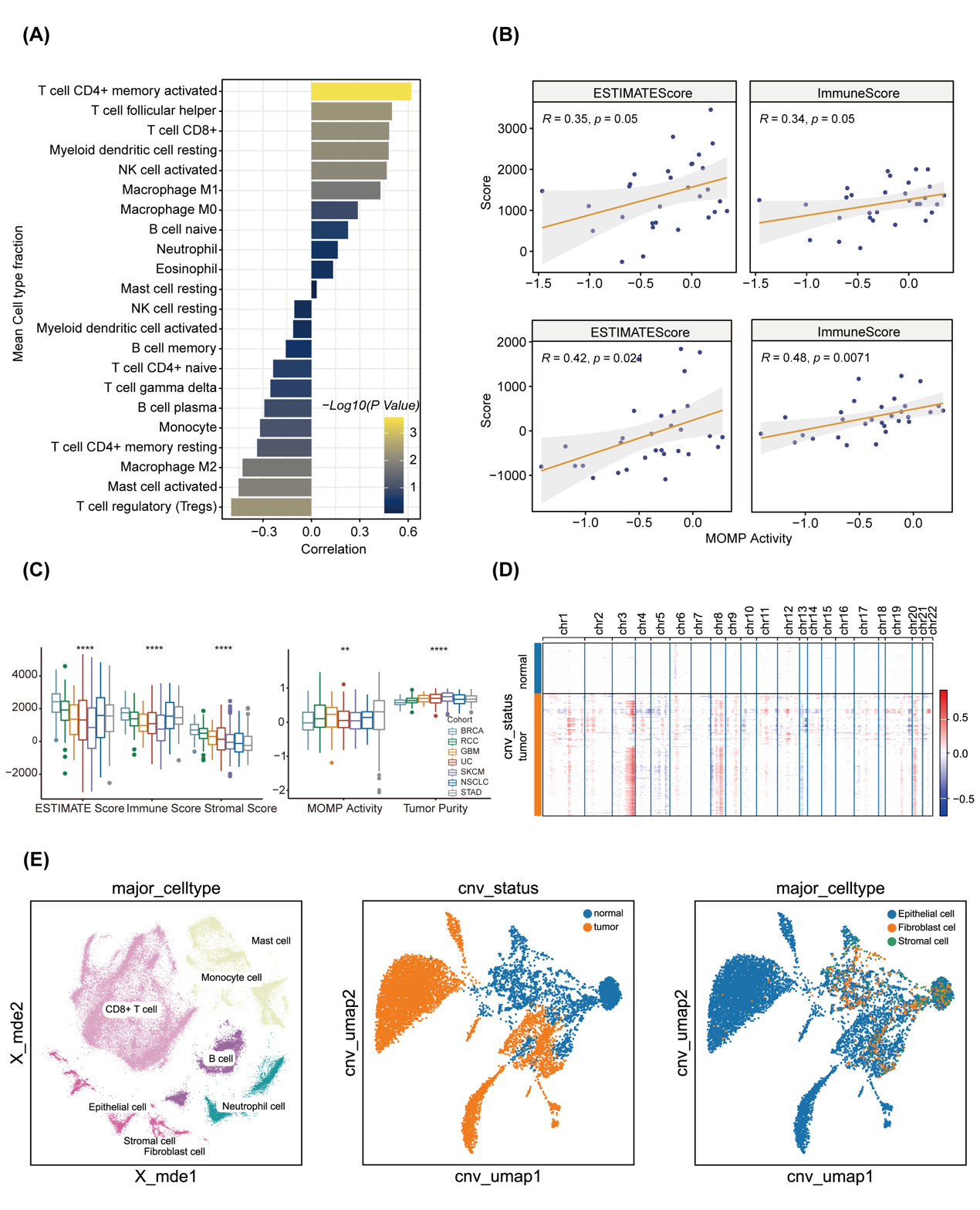
**(A) Correlation analysis of average MOMP activity with the fraction of immune cell infiltration across 30 solid tumors from TCGA by CIBERSORT algorithm. (B) Correlation analysis of average MOMP activity with ESTIMATE Score and Immune Score in low-purity (top) and high-purity (bottom) tumors from 30 solid tumor types in TCGA. (C) Distribution of ESTIMATE scores across different tumor types within the immunotherapy cohorts. (D) Heatmap displaying the copy number variation status in epithelial cells from NSCLC ICI scRNA-Seq. (E) NSCLC ICI scRNA-Seq cell type annotation and the distribution of copy number scores specifically in epithelial cells. MOMP, mitochondrial outer membrane permeabilization; TCGA, The Cancer Genome Atlas; NSCLC, non-small cell lung cancer; ICI, immune checkpoint inhibitor; scRNA-Seq, single cell RNA sequencing. Statistical significance was evaluated by a two-sided Wilcoxon rank sum test. **, p < 0.01; ****, p < 0.0001.

# FIGURE S3


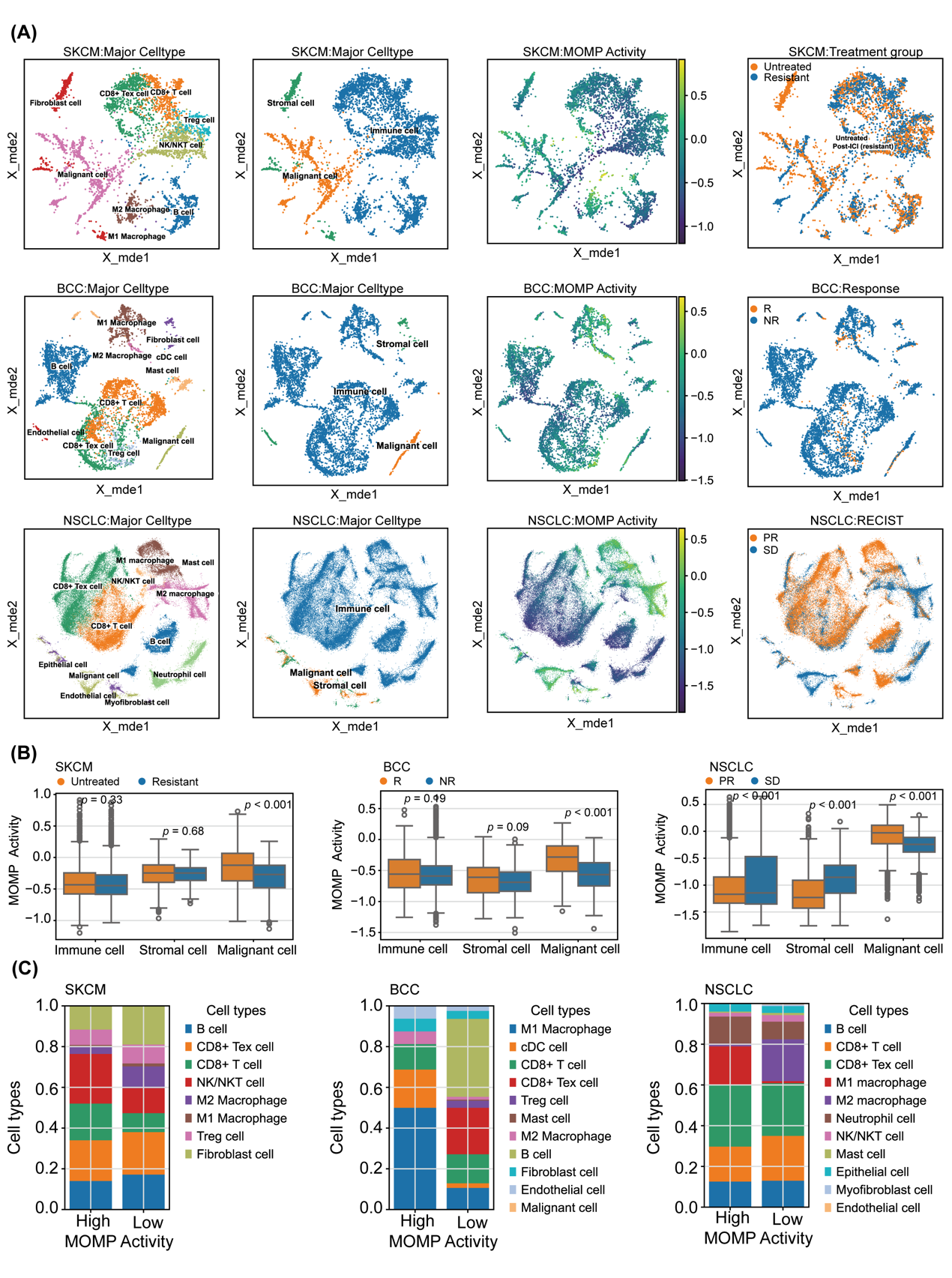
(A) Dimensionality reduction plots derived from ICI scRNA-Seq datasets for SKCM, BCC, and NSCLC illustrating the distribution of various cell types, MOMP activity levels, and responses to immunotherapy. (B) Boxplot analysis of MOMP activity in immune cells, stromal cells, and tumor cells within SKCM, BCC, and NSCLC ICI scRNA-Seq datasets, categorized by immunotherapy response. (C) Analysis of immune cell infiltration in patients stratified by tumor cell MOMP activity levels, examining the relationship between MOMP activity and the immune landscape within the tumor microenvironment. MOMP, mitochondrial outer membrane permeabilization; SKCM, skin cutaneous melanoma; BCC, basal cell cancer; NSCLC, non-small cell lung cancer; ICI, immune checkpoint inhibitor; scRNA-Seq, single cell RNA sequencing. Statistical significance was evaluated by a two-sided Wilcoxon rank sum test.

# FIGURE S4


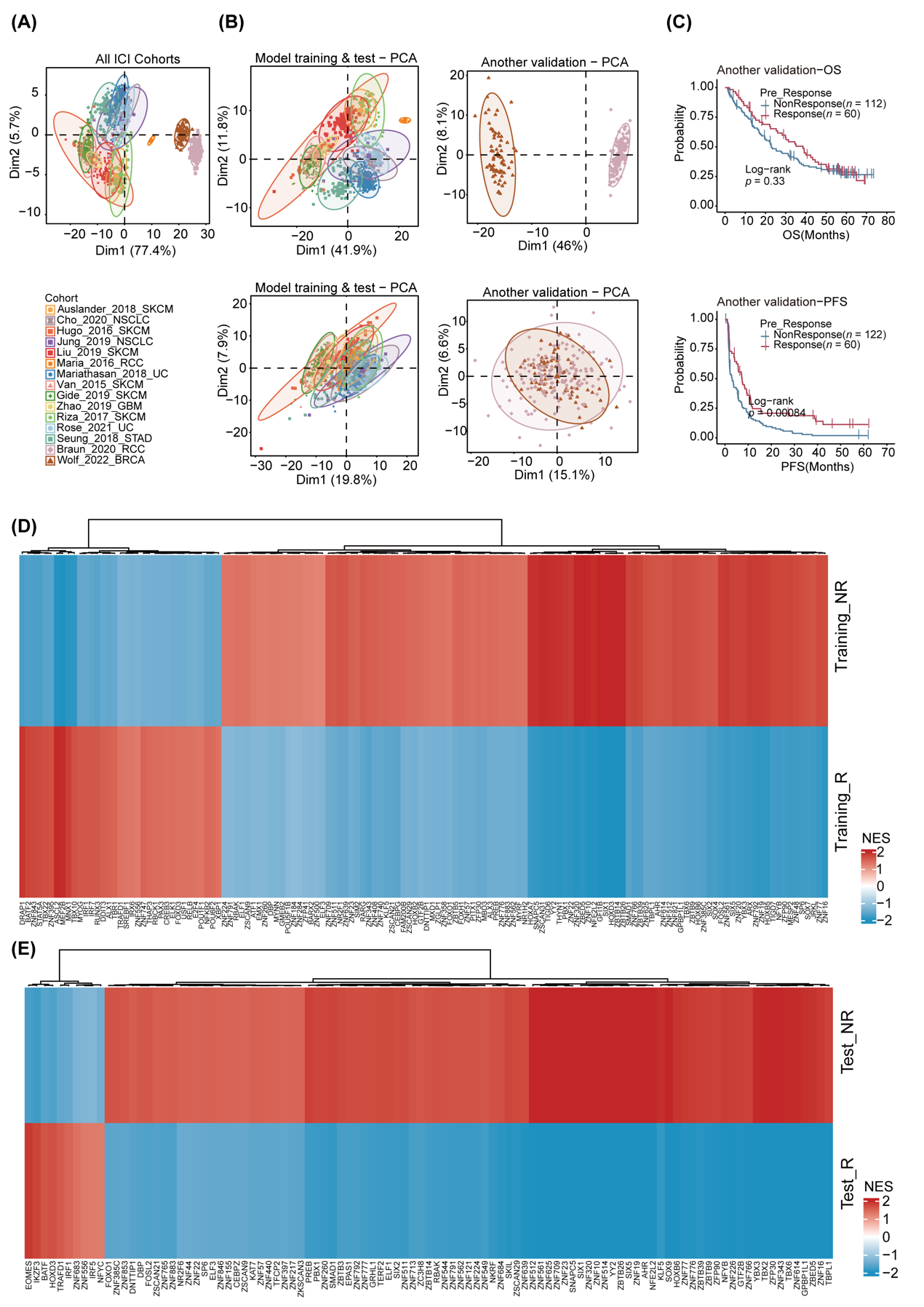
(A) Principal component analysis (PCA) plot illustrating the batch effect in all ICI RNA-Seq cohorts. (B) PCA plots of ICI RNA-Seq cohorts used for machine learning model training and testing, before and after batch effect removal (left); PCA plots of ICI RNA-Seq cohorts used for independent validation of the machine learning model, showcasing the data before and after batch effect mitigation (right). (C) Kaplan-Meier survival curves presenting OS and PFS stratified by ICI response predictions, as determined by the machine learning model, in another validation cohort. (D) MRs for immunotherapy responders and non-responders predicted accurately by the MOMP.Sig model in the training cohort. (E) MRs for immunotherapy responders and non-responders predicted accurately by the MOMP.Sig model in the test cohort. PCA, principal component analysis; ICI, immune checkpoint inhibitor; scRNA-Seq, single cell RNA sequencing; OS, overall survival; PFS, progression-free survival; MR, master regulators; NR, non-response; R, response.

# FIGURE S5

(A) ROC curve for assessing the predictive capability of FOXO1 expression levels for immunotherapy response in a pan-cancer ICI cohort
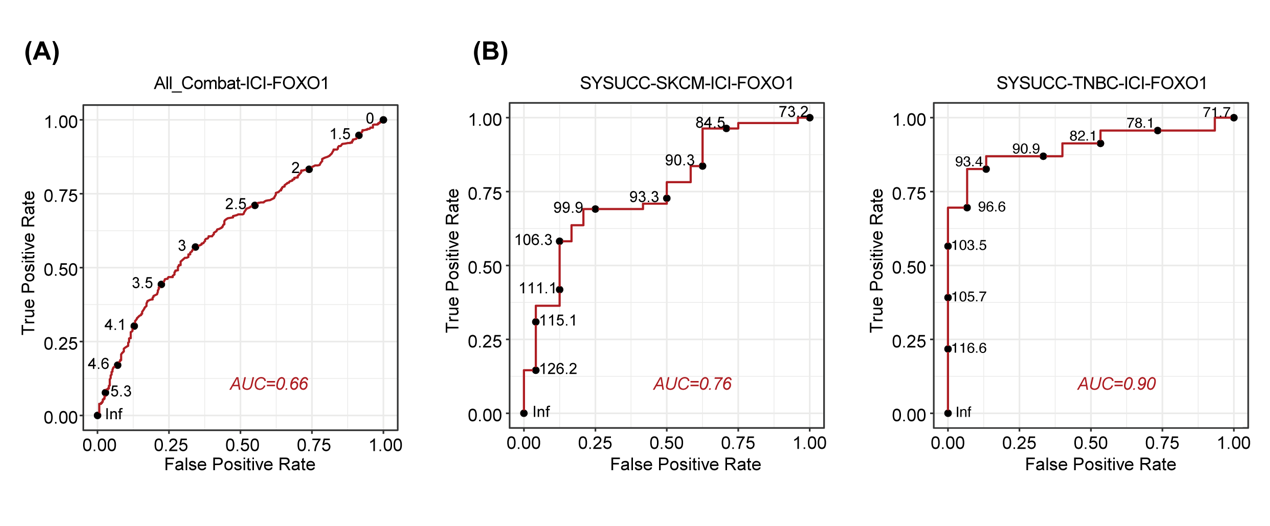
. (B) ROC curves evaluating the predictive power of the FOXO1 IHC H-score for immunotherapy response in SYSUCC-SKCM and TNBC cohorts. ROC, receiver operating characteristic; AUC, area under the ROC curve; IHC, immunohistochemistry; SYSUCC, Sun Yat-sen University Cancer Center; SKCM, skin cutaneous melanoma; TNBC, triple-negative breast cancer.
