# Supplementary material for: Comprehensive pan‐cancer analysis of mitochondrial outer membrane permeabilisation activity reveals positive immunomodulation and assists in identifying potential therapeutic targets for immunotherapy resistance
Source: Clin Transl Med. 2024 Jun 20;14(6):e1735. doi: 10.1002/ctm2.1735 (PMC11187817; doi:10.1002/ctm2.1735)
Supplement: Supplementary file 2 — Supporting information [file CTM2-14-e1735-s002.docx]

**Supplementary Tables**

[Table S1. MOMP activity gene sets 2](#_Toc165373369)

[Table S2. Pan-cancer ICI RNA-Seq cohorts 4](#_Toc165373370)

[Table S3. cell marker of ICI scRNA-Seq annotation 5](#_Toc165373371)

[Table S4. Pan-cancer scRNA-Seq datasets 6](#_Toc165373372)

[Table S5. Machine learning models from sklearn 8](#_Toc165373373)

[Table S6. SYSUCC-SKCM-ICI clinical information 9](#_Toc165373374)

[Table S7. SYSUCC-TNBC-ICI clinical information 10](#_Toc165373375)

[Table S8. qPCR Primers 11](#_Toc165373376)

[Table S9. Pan-cancer ICI predict signatures genes 12](#_Toc165373377)

[Table S10. Pan-cancer ICI predict signatures descriptions 15](#_Toc165373378)

[Table S11. MOMP activity-negative correlation genes and immunotherapy resistance MR 17](#_Toc165373379)

[Reference: 18](#_Toc165373380)

# Table S1. MOMP activity gene sets

| **Pathway** | **Gene** |
| --- | --- |
| Inflammasomes Pathway | AIM2,APP,BCL2,BCL2L1,CASP1,HMOX1,HSP90AB1,MEFV,NFKB1,NFKB2,NLRC4,NLRP1,NLRP3,P2RX7,PANX1,PSTPIP1,  PYCARD,RELA,SUGT1,TXN,TXNIP,CASP8,IL1B,MALT1,CTSB,NOX1,TLR4 |
| cGAS-STING Pathway | CGAS,DDX41,DTX4,IFI16,IRF3,MRE11,NLRC3,NLRP4,PRKDC,STAT6,STING1,TBK1,TREX1,TRIM21,XRCC5,XRCC6 |
| NF-kB Pathway | CHUK,FADD,IKBKB,IKBKG,IL1A,IL1R1,MAP3K1,MAP3K14,MAP3K7,MYD88,NFKB1,NFKBIA,RELA,RIPK1,TAB1,TNF,TNFAIP3,TNFRSF1A,TNFRSF1B,TRADD,TRAF6,BIRC2,BIRC3,BTRC, CD27,CD40,CD40LG,CD70,CUL1,EDA,EDA2R,EDAR,EDARADD,  FASLG,FBXW11,LTA,LTB,LTBR,NFKB2,PSMA1,PSMA2,PSMA3,PSMA4,PSMA5,PSMA6,PSMA7,PSMA8,PSMB1,PSMB10,PSMB11,PSMB2,PSMB3,PSMB4,PSMB5,PSMB6,PSMB7,PSMB8,PSMB9, PSMC1,PSMC2,PSMC3,PSMC4,PSMC5,PSMC6,PSMD1,PSMD10,  PSMD11,PSMD12,PSMD13,PSMD14,PSMD2,PSMD3,PSMD4,PSMD5,PSMD6,PSMD7,PSMD8,PSMD9,PSME1,PSME2,PSME3,  PSME4,PSMF1,RELB,RPS27A,SEM1,SKP1,TNFRSF11A,TNFRSF11B,TNFRSF12A,TNFRSF13B,TNFRSF13C,TNFRSF14,TNFRSF17,  TNFRSF18,TNFRSF25,TNFRSF4,TNFRSF6B,TNFRSF8,TNFRSF9,TNFSF11,TNFSF12,TNFSF13,TNFSF13B,TNFSF14,  TNFSF15,TNFSF18,TNFSF4,TNFSF8,TNFSF9,TRAF2,TRAF3,UBA3,UBA52,UBB,UBC,UBE2M,NFKBIE,REL |
| Apoptosis Pathway | AIFM1,AKT1,AKT2,AKT3,APAF1,ATM,BAD,BAX,BCL2,BCL2L1,BID,BIRC2,BIRC3,CAPN1,CAPN2,CASP10,CASP3,CASP6,CASP7,CASP8,CASP9,CFLAR,CHP1,CHP2,CHUK,CSF2RB,CYCS,DFFA,DFFB,ENDOD1,ENDOG,EXOG,FADD,FAS,FASLG,IKBKB,  IKBKG,IL1A,IL1B,IL1R1,IL1RAP,IL3,IL3RA,IRAK1,IRAK2,IRAK3,IRAK4,MAP3K14,MYD88,NFKB1, NFKBIA,PIK3CB,PIK3CD,PIK3CG,PIK3R1,PIK3R2,PIK3R3,PIK3R5,PPP3CA,PPP3CB,PPP3CC,PPP3R1,PPP3R2,PRKACA,PRKACB,  PRKACG,PRKAR1A,PRKAR1B,PRKAR2A,PRKAR2B,PRKX,RELA,RIPK1,TNF,TNFRSF10A,TNFRSF10B,TNFRSF10C,TNFRSF10D,  TNFRSF1A,TNFSF10,TP53,TRADD,TRAF2,XIAP,ACIN1,ADD1,APC,APIP,APPL1,ARHGAP10,AVEN,BAK1,BBC3,BCAP31,  BCL2L11,BMF,BMX,C1QBP,CARD8,CD14,CDH1,CDKN2A,CLSPN,CTNNB1,DAPK1,DAPK2,DAPK3,DBNL,DCC,DIABLO,DNM1L,  DSG1,DSG2,DSG3,DSP,DYNLL1,DYNLL2,E2F1,FNTA,GAS2,GSDMD,GSDME,GSN,GZMB,H1-0,H1-1,H1-2,H1-3,  H1-4,H1-5,HMGB1,HMGB2,KPNA1,KPNB1,LMNA,LMNB1,LY96,MAGED1,MAPK1,MAPK3,MAPK8,MAPT,NMT1,OCLN,OMA1,  OPA1,PAK2,PKP1,PLEC,PMAIP1,PPP1R13B,PRKCD,PRKCQ,PSMA1,PSMA2,PSMA3,PSMA4,PSMA5,PSMA6,PSMA7,PSMA8,  PSMB1,PSMB10,PSMB11,PSMB2,PSMB3,PSMB4,PSMB5,PSMB6,PSMB7,PSMB8,PSMB9,PSMC1,PSMC2,PSMC3,PSMC4,PSMC5,  PSMC6,PSMD1,PSMD10,PSMD11,PSMD12,PSMD13,PSMD14,PSMD2,PSMD3,PSMD4,PSMD5,PSMD6,PSMD7,  PSMD8,PSMD9,PSME1,PSME2,PSME3,PSME4,PSMF1,PTK2,ROCK1,RPS27A,SATB1,SEM1,SEPTIN4,SFN,SPTAN1,STAT3,STK24,  STK26,TFDP1,TFDP2,TICAM1,TICAM2,TJP1,TJP2,TLR4,TP53BP2,TP63,TP73,UACA,UBA52,UBB,UBC,UNC5A,UNC5B,VIM,YWHAB,YWHAE,YWHAG,YWHAH,YWHAQ,YWHAZ,BCL2L2,BIRC5,BNIP3L,BOK,CASP1,CASP2,CASP4,CRADD,HELLS,HRK,IGF1,IGF1R,IGF2,IRF1,IRF2,IRF3,IRF4,IRF5,IRF6,IRF7,JUN,LTA,MAP2K4,MAP3K1,MAPK10,MCL1,MDM2,MIR3191,MIR7846,MYC,NFKBIB,NFKBIE,PRF1,SCAF11,TNFRSF1B,TNFRSF21,TNFRSF25,TRAF1,TRAF3 |
| Pyroptosis Pathway | BAK1,BAX,CASP1,CASP3,CASP4,CASP5,CHMP2A,CHMP2B,CHMP3,CHMP4A,CHMP4B,CHMP4C,CHMP6,CHMP7,CYCS,ELANE,GSDMD,GSDME,GZMB,HMGB1,IL18,IL1A,IL1B,IRF1,IRF2,TP53,TP63 |

# Table S2. Pan-cancer ICI RNA-Seq cohorts

| **Cohort** | **Treatment** | **NR(SD/PD)** | **R(CR/PR)** | **Age** | **Gender** | **Stage** | **TMB** | **ITH** | **OS** | **PFS** |
| --- | --- | --- | --- | --- | --- | --- | --- | --- | --- | --- |
| Wolf_2022_BRCA^1^ | anti-PD-1 | 38 | 31 | - | - | - | - | - | - | - |
| Zhao_2019_GBM^2^ | anti-PD-1 | 17 | 17 | √ | - | - | - | - | √ | - |
| Seung_2018_STAD^3^ | anti-PD-1 | 57 | 21 | - | - | - | - | - | - | - |
| Cho_2020_NSCLC^4^ | anti-PD-1 | 11 | 5 | - | - | - | - | - | - | - |
| Jung_2019_NSCLC^5^ | anti-PD-1 | 19 | 8 | √ | √ | - | - | - | - | √ |
| Braun_2020_RCC^6^ | anti-PD-1 | 133 | 39 | √ | √ | √ | - | - | √ | √ |
| Maria_2016_RCC^7^ | anti-PD-1 | 7 | 4 | - | - | - | - | - | - | - |
| Mariathasan_2018_UC^8^ | anti-PD-1 | 230 | 68 | - | √ | - | - | - | √ | - |
| Rose_2021_UC^9^ | anti-PD-1 | 70 | 16 | √ | √ | √ | - | - | √ | √ |
| Rose_2021_UC | anti-PD-L1 | 3 | - | √ | √ | √ | - | - | √ | √ |
| Auslander_2018_SKCM^10^ | anti-CTLA-4 | 2 | - | √ | - | - | - | - | - | - |
| Auslander_2018_SKCM | anti-PD-1 | 17 | - | √ | - | - | - | - | - | - |
| Auslander_2018_SKCM | anti-PD-1+anti-CTLA-4 | - | 2 | √ | - | - | - | - | - | - |
| Hugo_2016_SKCM^11^ | anti-PD-1 | 13 | 15 | √ | √ | √ | √ | - | √ | - |
| Liu_2019_SKCM^12^ | anti-PD-1 | 30 | 21 | - | - | √ | - | - | √ | √ |
| Liu_2019_SKCM | anti-PD-1 | 42 | 28 | - | - | √ | - | - | √ | √ |
| Gide_2019_SKCM^13^ | anti-CTLA-4+  anti-PD-1 | 15 | 26 | √ | √ | - | - | - | √ | - |
| Gide_2019_SKCM | anti-PD-1 | 27 | 23 | √ | √ | - | - | - | √ | - |
| Riza_2017_SKCM^14^ | anti-PD-1 | 39 | 10 | - | - | - | - | - | √ | - |
| Van_2015_SKCM^15^ | anti-PD-1 | 90 | 63 | - | √ | √ | - | - | √ | - |

Abbreviation: R: Responder; NR: Non-responder; BRCA: Breast cancer; GBM: Glioblastoma; STAD: Stomach cancer; NSCLC: Non-small cell lung cancer; RCC: Renal cell carcinoma; UC: Urothelial carcinoma; SKCM: Skin cutaneous melanoma; TMB: Tumor mutational burden; ITH: Intratumor heterogeneity.

# Table S3. cell marker of ICI scRNA-Seq annotation

| **Cell type** | **SKCM/BCC marker genes** | **NSCLC marker genes** |
| --- | --- | --- |
| B cell | CD19, CD79A, MS4A1, CD20 | CD19, CD79A, MS4A1, CD20 |
| CD8+T cell | CD8A, CD8B, CD8, CD3, CXCR5 | CD8A, CD3E, CD3D, CCL5 |
| CD8+Tex cell | CD279, CTLA4, BTLA, PDCD1, HNF1A, TCF1 | BTLA, GZMK, LAG3, NKG7, TIGIT |
| Treg | CCR4, FOXP3, CTLA4 | FOXP3, CD40LG, CTLA4 |
| CD4+T cell | CD11C, CD3, CD4 | CCR7, CD3D, CD4 |
| NK/NKT cell | CD3, CD28, CD161 | CD8A, FCER1G, TYROBP |
| Mast cell | TPSAB1, CPA3, TPSB2 | TPSAB1, CPA3, TPSB2, KIT |
| cDC cell | IRF8, CD4, CD141, CD1C, CLEC10A | CCL19, CCL22, CCR7, CLEC9A, EBI3 |
| pDC cell | CD123, CLEC4C | LILRA4, CLEC4C, IL3RA |
| M1 macrophage | CD38, CD169, CXCL9, CXCL10, FOS, S100A8 | CCL5, CCR7, CXCL9, CXCL10, CD68 |
| M2 macrophage | ANXA1, CD163, CD204,  CD206, MERTK, MRC1 | ARG1, CD163, CCL18, CD206 |
| Neutrophil | - | CSF3R, CD11B, CD15, CD16 |
| Endothelial cell | CD31, PECAM1, VWF, CDH5, CLDN5 | CD31, PECAM1, VWF, CDH5,  CLDN5 |
| Fibroblast cell | COL1A1, DCN, FN1, APOD, CD90 | COL1A1, CD163, CD90 |
| Myofibroblasts | ACTA2, CCL21, ENG | ACTA2, LUM, MYLK |
| Malignant cell | HMB45, SOX10, CD146, CD36, DCT, LDH, KRT14, PTCH1, PTCH2 | EGFR, EPCAM, TTF1, ALK |

All types of cell marker genes are sourced from the Cellmarker database.^16^

# Table S4. Pan-cancer scRNA-Seq datasets

| **Abbreviation** | **GEO numbers** | **Location** | **Location_Num** |
| --- | --- | --- | --- |
| BCC | GSE141526 | Skin | 5 |
| BRCA | EMTAB8107 | Breast | 6 |
| BRCA | GSE143423 | Breast | 6 |
| BRCA | GSE148673 | Breast | 6 |
| BRCA | GSE150660 | Breast | 6 |
| BRCA | GSE161529 | Breast | 6 |
| BRCA | GSE176078 | Breast | 6 |
| CESC | GSE168652 | Pelvic cavity | 8 |
| CHOL | GSE125449 | Liver | 7 |
| CHOL | GSE138709 | Liver | 7 |
| CRC | EMTAB8107 | Colorectum | 3 |
| CRC | GSE146771 | Colorectum | 3 |
| CRC | GSE166555 | Colorectum | 3 |
| ESCA | GSE160269 | Esophagus | 1 |
| GIST | GSE162115 | Soft tissue | 3 |
| Glioma | GSE131928 | Brain | 10 |
| Glioma | GSE138794 | Brain | 10 |
| Glioma | GSE139448 | Brain | 10 |
| Glioma | GSE141383 | Brain | 10 |
| Glioma | GSE141460 | Brain | 10 |
| Glioma | GSE141982 | Brain | 10 |
| Glioma | GSE148842 | Brain | 10 |
| Glioma | GSE84465 | Brain | 10 |
| HB | GSE180665 | Liver | 7 |
| HNSC | GSE103322 | Head & Neck | 2 |
| KICH | GSE159115 | Kidney | 2 |
| KIRC | GSE171306 | Kidney | 2 |
| LIHC | GSE125449 | Liver | 7 |
| LIHC | GSE146115 | Liver | 7 |
| LIHC | GSE146409 | Liver | 7 |
| LIHC | GSE166635 | Liver | 7 |
| MB | GSE119926 | Brain | 10 |
| MB | GSE155446 | Brain | 10 |
| MCC | GSE117988 | Skin | 5 |
| MCC | GSE118056 | Skin | 5 |
| NSCLC | EMTAB6149 | Lung | 7 |
| NSCLC | GSE117570 | Lung | 7 |
| NSCLC | GSE127465 | Lung | 7 |
| NSCLC | GSE143423 | Lung | 7 |
| NSCLC | GSE148071 | Lung | 7 |
| NSCLC | GSE150660 | Lung | 7 |
| OS | GSE162454 | Bone | 1 |
| OV | EMTAB8107 | Pelvic cavity | 8 |
| OV | GSE118828 | Pelvic cavity | 8 |
| OV | GSE130000 | Pelvic cavity | 8 |
| OV | GSE147082 | Pelvic cavity | 8 |
| OV | GSE151214 | Pelvic cavity | 8 |
| OV | GSE154600 | Pelvic cavity | 8 |
| OV | GSE158722 | Pelvic cavity | 8 |
| PAAD | CRA001160 | Pancreas | 7 |
| PAAD | GSE111672 | Pancreas | 7 |
| PAAD | GSE141017 | Pancreas | 7 |
| PAAD | GSE148673 | Pancreas | 7 |
| PAAD | GSE154778 | Pancreas | 7 |
| PAAD | GSE162708 | Pancreas | 7 |
| PAAD | GSE165399 | Pancreas | 7 |
| PPB | GSE163678 | Soft tissue | 3 |
| PRAD | GSE137829 | Prostate | 4 |
| PRAD | GSE141445 | Prostate | 4 |
| PRAD | GSE143791 | Prostate | 4 |
| PRAD | GSE176031 | Prostate | 4 |
| SCLC | GSE150766 | Lung | 7 |
| SKCM | GSE115978 | Skin | 5 |
| SKCM | GSE72056 | Skin | 5 |
| SS | GSE131309 | Soft tissue | 3 |
| STAD | GSE134520 | Stomach | 1 |
| THCA | GSE148673 | Head & Neck | 2 |

All single-cell data for various tumor types are sourced from the TISCH database (<http://tisch.comp-genomics.org>), and these data have been annotated with cell type information.

# Table S5. Machine learning models from sklearn

| **Abbreviation** | **Model names** | **Model categories** |
| --- | --- | --- |
| Logistic | Logistic Regression | Linear Models |
| SGDClassifier | Stochastic Gradient descent Classifier | Stochastic Gradient descent |
| SVC | Support Vector Classification | Support Vector Machines |
| NuSVC | Nu-Support Vector Classification | Support Vector Machines |
| KNN | K-Nearest Neighbors Classification | K-Nearest Neighbors |
| RadiusNN | Radius-based Neighbors Classification | K-Nearest Neighbors |
| DecisionTree | Decision Tree Classifier | Decision Trees |
| GradientBoost | Gradient Boosting Classifier | Gradient Boosting |
| HistGradientBoost | Histogram-Based Boosting Classifier | Gradient Boosting |
| RandomForest | Random Forest Classifier | Random Forests |
| ExtraTrees | Extremely Randomized Trees Classifier | Random Forests |
| MLP | Multi-layer Perceptron classifier | Neural Network |
| GaussianProcess | Gaussian process classification | Gaussian Process |

All machine learning models are source from sklearn (<https://scikit-learn.org/stable/>).

# Table S6. SYSUCC-SKCM-ICI clinical information

| **Characteristics** | **FOXO1 High**  **N=43 (%)** | **FOXO1 Low**  **N=36 (%)** | **P value** |
| --- | --- | --- | --- |
| **Age** |  |  | **0.656** |
| ＜60 | 33 (76.7) | 30 (83.3) |  |
| ≥60 | 10 (23.3) | 6 (16.7) |  |
| **Gender** |  |  | **1.000** |
| Male | 21 (48.8) | 18 (50) |  |
| Female | 22 (51.2) | 18 (50) |  |
| **PS** |  |  | **0.323** |
| 0 | 35 (81.4) | 33 (91.6) |  |
| 1-2 | 8 (18.6) | 3 (8.4) |  |
| **Subtype** |  |  | **0.456** |
| Cutaneous | 20 (46.5) | 14 (38.8) |  |
| Acral | 10 (23.3) | 13 (36.2) |  |
| Mucosal | 13 (30.2) | 9 (25) |  |
| **Tumor stage** |  |  | **1.000** |
| Stage III | 4 (9.3) | 3 (8.4) |  |
| Stage IV | 39 (90.7) | 33 (91.6) |  |
| **Liver metastasis** |  |  | **0.846** |
| No | 34 (79.1) | 30 (83.3) |  |
| Yes | 9 (20.9) | 6 (16.7) |  |
| **Brain metastasis** |  |  | **0.498** |
| No | 37 (86.1) | 33 (91.6) |  |
| Yes | 6 (13.9) | 3 (8.4) |  |
| **Type of immunotherapy** |  |  | **0.226** |
| Anti-PD-1/PD-L1/CTLA4 monotherapy | 17 (39.5) | 8 (22.2) |  |
| Anti-PD-1/PD-L1+ Anti-CTLA-4 combination | 3 (6.9) | 2 (5.5) |  |
| Anti-PD-1/PD-L1+ others combination | 23 (53.5) | 26 (72.2) |  |
| **OS events** | 33 | 21 | - |
| **Median** **OS** | 13.8(±5.8) | 30.8(±7.3) | **0.012** |
| **PFS events** | 41 | 29 | **-** |
| **Median PFS** | 3.8(±2.6) | 11.8(±5.1) | **0.0007** |
| **IHC FOXO1 H-Score** |  |  | **<0.0001** |
|  | 117.7(±15.1) | 89(±5.7) |  |
| **Response** |  |  | **<0.0001** |
| CR | 4 (9.3) | 3 (8.4) |  |
| PR | 1 (2.3) | 16 (44.5) |  |
| SD | 26 (60.5) | 6 (16.6) |  |
| PD | 12 (27.9) | 11 (30.5) |  |

# Table S7. SYSUCC-TNBC-ICI clinical information

| **Characteristics** | **FOXO1 High N=20 (%)** | **FOXO1 Low N=18 (%)** | **P value** |
| --- | --- | --- | --- |
| **Age** |  |  | **1.000** |
| ＜50 | 12 (60) | 11 (61.1) |  |
| ≥50 | 8 (40) | 7 (38.9) |  |
| **Menopause** |  |  | **1.000** |
| Yes | 9 (45) | 8 (40) |  |
| No | 11 (55) | 10 (60) |  |
| **BMI** |  |  |  |
|  | 23.2(±3.9) | 22.3(±1.6) | **0.476** |
| Ki67 |  |  |  |
|  | 0.616(±0.22) | 0.607(±0.21) |  |
| **Tumor stage** |  |  | **0.568** |
| Stage IIA-B | 5 (25) | 7 (38.8) |  |
| Stage IIIA-C | 15 (75) | 11 (61.2) |  |
| **Type of ICI** |  |  | **1.000** |
| Anti-PD-1/PD-L1+ chemotherapy | 20 (100) | 18 (100) |  |
| **ICI course** |  |  | **0.687** |
| ≤ 4 | 9 (45) | 6 (33.3) |  |
| > 4 | 11 (55) | 12 (66.7) |  |
| **IHC FOXO1 H-Score** |  |  | **<0.0001** |
|  | 108.6(±20.1) | 86.7(±8.7) |  |
| **Response** |  |  | **0.02** |
| R | 4 (20) | 11 (61.1) |  |
| NR | 16 (80) | 7 (38.9) |  |

# Table S8. qPCR Primers

| **Primers** | **Sequence (5'to3')** |
| --- | --- |
| BAK1-F | GTTTTCCGCAGCTACGTTTTT |
| BAK1-R | GCAGAGGTAAGGTGACCATCTC |
| BAX-F | CCCGAGAGGTCTTTTTCCGAG |
| BAX-R | CCAGCCCATGATGGTTCTGAT |
| GSDMD-F | GTGTGTCAACCTGTCTATCAAGG |
| GSDMD-R | CATGGCATCGTAGAAGTGGAAG |
| CASP3-F | CATGGAAGCGAATCAATGGACT |
| CASP3-R | CTGTACCAGACCGAGATGTCA |
| NLRP3-F | GATCTTCGCTGCGATCAACAG |
| NLRP3-R | CGTGCATTATCTGAACCCCAC |
| RELB-F | CAGCCTCGTGGGGAAAGAC |
| RELB-R | GCCCAGGTTGTTAAAACTGTGC |
| TBK1-F | TGGGTGGAATGAATCATCTACGA |
| TBK1-R | GCTGCACCAAAATCTGTGAGT |
| STING-F | CACTTGGATGCTTGCCCTC |
| STING-R | GCCACGTTGAAATTCCCTTTTT |
| IRF3-F | AGAGGCTCGTGATGGTCAAG |
| IRF3-R | AGGTCCACAGTATTCTCCAGG |
| CCL4-F | CTGTGCTGATCCCAGTGAATC |
| CCL4-R | TCAGTTCAGTTCCAGGTCATACA |
| CD274-F | TGGCATTTGCTGAACGCATTT |
| CD274-R | TGCAGCCAGGTCTAATTGTTTT |
| VEGFC-F | GAGGAGCAGTTACGGTCTGTG |
| VEGFC-R | TCCTTTCCTTAGCTGACACTTGT |
| FOXO1-F | GGATGTGCATTCTATGGTGTACC |
| FOXO1-R | TTTCGGGATTGCTTATCTCAGAC |
| GAPDH-F | CTGGGCTACACTGAGCACC |
| GAPDH-R | AAGTGGTCGTTGAGGGCAATG |

# Table S9. Pan-cancer ICI predict signatures genes

| **Signatures** | **Genes** |
| --- | --- |
| Cytotoxic.Sig | GZMA,PRF1 |
| PDL1.Sig | PDL1,PDCD1 |
| CD8.Sig | CD8A,CD8B,CD3D,CD3E,CD3G |
| TRS.Sig | CTLA4,CXCR6,LYST,CD38,GBP2,HLA-DRB5 |
| IFNG.Sig | IFNG,STAT1,IDO1,CXCL10,CXCL9,HLA-DRA |
| CRMA.Sig | CSAG1,CSAG2,CSAG3,MAGEA2,MAGEA2B,MAGEA3,MAGEA6,MAGEA12 |
| IRG.Sig | LEPR,PRLHR,NR2F2,PRL,NRP1,ANGPTL5,IGF1,TNFRSF10B,TNFRSF10A,PLAU,IFI30 |
| EMT.Sig | CDH1,CDH3,CLDN4,EPCAM,ST14,MAL2,VIM,SNAI2,ZEB2,FN1,MMP2,AGER |
| LRRC15.CAF.Sig | MMP11,COL11A1,C1QTNF3,CTHRC1,COL12A1,COL10A1,COL5A2,GJB2,THBS2,AEBP1,MFAP2,LRRC15,PLAU,ITGA11 |
| T.cell.inflamed.Sig | CD3D,IDO1,CIITA,CD3E,CCL5,GZMK,CD2,HLA-DRA,CXCL13,IL2RG,NKG7,HLA-E,CXCR6,LAG3,TAGAP,CXCL10,STAT1,GZMB |
| IMS.Sig | FAP,PDGFRB,CD163,CD169,SIGLEC1,IL10,CCL2,CCL8,CCL13,INHBA,VCAN,AXL,TWIST2,ADAM12,COL6A3,  STC1,ISG15,BCAT1,OLFML2B |
| Inflammatory.Sig | CCL5,CCR5,PDL1,CD3D,CD3E,CD8A,CIITA,CTLA4,CXCL10,CXCL11,CXCL13,CXCL9,GZMA,GZMB,HLA-DRA,HKA.DRB1,  HLA-E,IDO1,IL2RG,ITGAL,LAG3,NKG7,PDCD1,PRF1,PTPRC,STAT1,TAGAP |
| NLRP3.Sig | ARRDC1-AS1,CARD8,GSDMD,ATAT1,CD36,CPTP,DHX33,EIF2AK2,GBP5,NLRC3,PYDC2,SIRT2,TLR4,TLR6,USP50,  APP,CASP1,HSP90AB1,MEFV,NFKB1,NFKB2,NLRP3,P2RX7,PANX1,PSTPIP1,PYCARD,RELA,SUGT1,TXN,TXNIP |
| ImmmunCells.Sig | JMJD7,TRAF3IP2,UBE2C,CDCA5,TM4SF19,CLNK,TMEM171,CLDN7,CR2,SMEK3P,SPC24,CILP2,SYT6,ENTHD1,PRUNE2,  ALDH1L2,STOML3,NUDT10,KLHDC8B,FBLN1,FBLN2,C6orf223,FOXI1,FMOD,FOLR2,LYSMD2,ASPM,NUPR1,PPA2,  GPR31,GRIA1,GRM7,APBB2,BIRC5,APOC2,ITGA3,KRT4,LALBA,MATN3,MFAP2,SCGB2A2,MKI67,MMP12,MT1G,MUSK,MYL1,  CEACAM6,ROR1,LEF1,DUSP13,ZNF219,RASL12,TREM2,CYTL1,MXRA8,MAP2K5,PRPH,CD177,TSHZ3,RNASE1,RRM2,CCL18,  SEPP1,SPP1,STC1,TK1,TRPC4,TYMS,CACNG1,KIRREL2,TEAD2,MAEL,STC2,ADAM21,DLK1,SLC16A3,PKDCC,KIAA0101,CDH1,  ARSF,CD244,CRTAM,GBP1P1,GIMAP4,KIR2DL4,LINC00243,MYO1G,OTOF,SH2D2A,SHC3,SPATA13,TDRD15,TUBA8,RPL36AP41,  MMP9,UNC80,NACA2,ZNF462,CORO7,NACA3P,DHRS9,ASAH2,GDF1,ZNF610,PLA2G2D,EIF4A2,RIMS2,ZNF880 |
| TcellExc.Sig | AHCY,APP,ATP5D,ATP5G3,BOP1,BTF3,BZW2,C17orf76-AS1,C19orf48,C1QBP,C6orf48,CACYBP,CCT3,CCT4,CCT6A,CCT7,  CDCA7,CDK4,CHCHD2,CTPS1,DARS,DCTPP1,DDX21,EEF1B2,EEF1D,EEF1G,EEF2,EIF2S3,EIF3E,EIF3F,EIF3G,EIF3K,EIF3M,  EIF4A1,ENO1,EXOSC5,FAM92A1,FARSA,FBL,FKBP4,GAS5,GGH,GNB2L1,GNL3,GPATCH4,GPI,HMGB1,HNRNPA1,HNRNPC,  HNRNPH1,HNRNPM,HSPD1,IDH2,IFRD2,ILF2,ILF3,IMPDH2,ISYNA1,LDHB,LSM4,LSM7,LYPLA1,MAGEC1,MCM7,MDH2,  MKI67IP,MRPL15,MRPL37,MRPL4,MRPS12,NACA,NCL,NDUFA11,NME1,NME2,NOLC1,NOP16,NPM1,NREP,PABPC1,PAICS,  PFN1,PLEKHJ1,POLD2,POLR1D,POLR2E,PPA1,PPIA,PRMT1,PTMA,PUF60,RPL10,RPL10A,RPL11,RPL12,RPL13,RPL13A,RPL13AP5,  RPL14,RPL15,RPL17,RPL18,RPL18A,RPL19,RPL21,RPL22,RPL26,RPL27,RPL27A,RPL28,RPL29,RPL3,RPL30,RPL31,RPL32,RPL35,  RPL36,RPL36A,RPL37,RPL37A,RPL39,RPL4,RPL41,RPL5,RPL6,RPL7,RPL7A,RPL8,RPL9,RPLP0,RPLP1,RPLP2,RPS10,RPS11,RPS13,  RPS14,RPS15,RPS15A,RPS16,RPS17,RPS17L,RPS18,RPS19,RPS2,RPS20,RPS21,RPS23,RPS24,RPS25,RPS27,RPS27A,RPS28,RPS3,  RPS3A,RPS4X,RPS5,RPS6,RPS7,RPS8,RPS9,RPSA,RQCD1,RRS1,RSL1D1,RUVBL2,SAE1,SERBP1,SERPINF1,SET,SHMT2,SLC19A1,  SLC25A13,SLC25A6,SMARCA4,SMIM15,SNHG15,SNHG6,SNRPB,SNRPC,SNRPD1,SNRPD2,SNRPE,SOX4,SRM,SSB,SSR2,TIMM13,  TIMM44,TIMM50,TOP1MT,TPI1,TRAP1,TRIM28,TUBB,TYMS,UBA52,UCK2,UHRF1,UQCRFS1,UQCRH,VDAC2,XIST,ZFAS1 |
| MOMP.Sig | SDC4,TSPAN3,EPCAM,MRPS18A,LGALS1,PGRMC1,RNASE4,MANBAL,PLPP2,LGALS3BP,PRDX5,AGR2,CALML5,PDLIM1,TM4SF1,  SCCPDH,RAC1,NQO1,TUBA1B,TSPAN8,DEFB1,DST,DSTN,TMEM106C,DUSP4,MACC1,TSPAN1,KLF4,TMEM45B,ANXA4,CYB5A,  EDN1,HSPB1,SPTSSB,S100P,TACSTD2,ENO1,IFITM3,EIF6,PHLDA3,SEC61G,NDUFA4,ANXA2,PON2,CD47,IDH1,MYO1C,POLR1C,  SPINT2,TMEM9,CLDN7,DHCR24,PKM,MAD2L1BP,RND3,S100A16,S100A6,AGR3,MTCH1,CD9,ILF2,TPI1,PSMB5,SERPINA1,GGH,  SLC25A5,GPX2,PERP,RBPMS,CRABP2,S100A14,CCT5,JPT1,CLDN3,PPA1,SDR16C5,RHOC,KRT8,KIF5B,ANXA3,MGST1,TXN,  TNFRSF12A,C6orf132,RNF128,AKR1B1,HES4,TUBB,RRP36,BASP1,GPRC5A,KRT6A,CLDN4,NCOA7,TSPAN13,WFDC2,RANBP1,  LYPD6B,PRDX2,EFNB2,TMEM176B,TMEM176A,CAPN8,INHBB,SLC44A4,RAB25,GSTP1,SUMO2,FH,LURAP1L,ARHGAP29,THSD4,  GDF15,YWHAQ,PTMA,ETHE1,CLDND1,HSP90AB1,LCN2,MAL2,RAB20,ATP1B1,IRS2,TRIM29,SFN,NPW,NOXO1,NNMT,NPM1,ODC1,  PGK1,EI24,REXO2,LAPTM4B,CLCA2,KRT17,SNRPB,EMP2,PAPOLA,ERH,TCEAL8,F11R,KRT19,TOMM34,EMP1,EIF3K,LSM4,  CEACAM6,LMNA,TTC9,PSMD8,NUCKS1,TPM1,PAK1,COX6A1,PPDPF,LDHB,ECHS1,TNFRSF21,MRPL51,TMEM41A,CDH1,CD151,NXN,  RAB13,VIM,PLK2,ZFP36L1,PDCD5,SERPINB5,CCT6A,CACYBP,CD59,YBX1,YIPF3,REG4,S100A13,FDFT1,HNRNPAB,STMN1,RUVBL1,  CCND1,FHL2,CMTM6,VSIG2,LDHA,PDZK1IP1,HES2,KRT10,KRT5,KCTD14,COX6B1,GPX4,MSLN,PSMB2,NDUFB11,SRD5A3,CRK,  VKORC1,GJB1,TSTA3,SNRPB2,RCN1,UQCR11,NEK6,YWHAE,PHLDA2,C6orf89,IGFBP3,CYC1,OCIAD2,RASL10A,RAN,CCDC124,  TMSB15A,KRT18,PTTG1,S100A10,NES,TIMM22,ARL2,RARRES1,CMTM7,PHLDA1,MARCKSL1 |

# Table S10. Pan-cancer ICI predict signatures descriptions

| **Category** | **DOI** | **Description** |
| --- | --- | --- |
| CD8 | 10.1038/s41591-018-0136-1 | Gene expression level of CD8A + CD8B + CD3D + CD3E + CD3G |
| CRMA | 10.1016/j.cell.2018.03.026 | Anti-CTLA4 resistance MAGE genes, including MAGEA2, MAGEA2B, MAGEA3, MAGEA6, and MAGEA12 |
| Cytotoxic | 10.1016/j.cell.2014.12.033 | Molecular and genetic properties of tumors associated with local immune cytolytic activity |
| EMT | 10.1016/j.lungcan.2019.10.012 | A gene expression signature of 12 epithelial-to-mesenchymal transition (EMT)  related genes that predicted response to immune checkpoint blockade in lung cancer |
| ImmmunCells | 10.1038/s41467-020-18546-x | A gene expression signature of TREM2hi macrophages and γδ T cells predicts immunotherapyresponse |
| IMPRES | 10.1038/s41591-018-0157-9 | Immuno-predictive score (IMPRES), a predictor of Immune checkpoint blockade (ICB) response in  melanoma based on 28 immune checkpoint genes |
| IMS | 10.1038/s41525-021-00169-w | Ratio of the interferon-γ signature to the immunosuppression signature predicts anti-PD-1  therapy response in melanoma |
| IFNG | 10.1172/JCI91190 | Interferon gamma (IFNγ) response biomarkers of 6 genes including IFNG, STAT1, IDO1, CXCL10,  CXCL9, and HLA-DRA |
| Inflammatory | 10.1016/j.lungcan.2019.10.012 | A gene expression signature of 27 inflammation related genes that predicted response to immune  checkpoint blockade in lung cancer |
| IPRES | 10.1016/j.cell.2016.02.065 | IPRES (innate anti-PD-1 resistance) that included 16 genes involved in cell adhesion, extracellular  matrix remodeling, angiogenesis, wound healing, and mesenchymal transition that predicted response  to anti-PD-1 antibody therapy in melanoma |
| IRG | 10.1080/2162402X.2019.1659094 | A prognostic signature based on 11 immune-related genes (IRGs) for predicting CC (cervical cancer)  patients’ response to immune checkpoint inhibitors |
| LRRC15.CAF | 10.1158/2159-8290.CD-19-0644 | A signature of 14 marker genes of a specific type of carcinoma-associated fibroblasts (CAF)  –“LRRC15+ CAFs” that correlated with poor response to anti-PD-L1 therapy |
| MOMP |  | The tumor cell MOMP signature identified in this study |
| NLRP3 | 10.1093/bib/bbaa345 | ssGSEA score based NLRP3-inflammasome-related genes |
| PDL1 | 10.1056/NEJMoa1200690 | Gene expression level of PD-L1 + PD-L2 + PD-1 |
| T.cell.inflamed | 10.1172/JCI91190 | An 18 gene “T-cell–inflamed gene expression signature” that can predict clinical benefit of anti-PD-1 in various cancer types (melanoma,head and neck squamous cell carcinomas, digestive cancers, ovarian and triple negative breast cancers) |
| TcellExc | 10.1016/j.cell.2018.09.006 | A Cancer Cell Program Promotes T Cell Exclusion and Resistance to Checkpoint Blockade |

# Table S11. MOMP activity-negative correlation genes and immunotherapy resistance MR

| **Cohort** | **Gene** |
| --- | --- |
| ICI resistance MRs in  Training set | ZGLP1,PREB,SNAPC5,CEBPG,SIX2,DNTTIP1,ZNF511,ZNF57,ZNF792,ZNF358,MESP2,ZSCAN29,ZFP90,SIX5,DBP,ZNF320,ZNF791,  ZNF709,ZNF883,ARX,AHR,ZNF48,ELF1,ZNF385C,EMX1,ZBTB9,ZFP30,FOXO1,FOSL2,ALX3,GMEB2,ZNF740,ZNF776,FAM200B,  ZFP41,THYN1,GTF2B,HOXA7,HOXB2,HOXB5,HOXB6,HOXD3,ZNF260,ZNF284,YY2,MXD1,SMAD1,ATF1,NFE2L2,NFYB,PBX1,  ZNF639,PITX1,MBD3,POU5F1B,ZNF562,ZNF821,MYNN,ZBTB26,RBAK,ZBED5,ZNF77,RARG,GPBP1L1,ZSCAN31,SIM2,SIX1,  SOX4,KLF5,TBX6,NR2F1,NR1H2,MYRF,ZBTB14,ZNF10,ZNF16,ZNF19,ZNF20,ZNF22,ZNF23,ZSCAN21,ZNF184,ZSCAN9,ZNF224,  ZNF343,ZNF408,ZNF614,SP6,TIGD6,GFI1B,SOX7,ZNF512,YBX3,BARX2,JRKL,FOXH1,ZNF766,ZNF625,TIGD7,ZNF561,TBPL1,  ZBTB39,ZBTB5 |
| ICI resistance MRs in  Test set | PREB,SNAPC5,SIX2,KAT7,DNTTIP1,ZNF511,ZNF440,ZNF57,ZNF792,ZNF684,ZSCAN29,ZFP90,SIX5,DBP,ZNF320,ZNF791,  ZNF709,ZNF883,AHR,ELF1,ELF3,ZNF385C,EPAS1,NR2F6,ZBTB9,ZFP30,FOXO1,FOSL2,ZNF549,ZNF777,ZNF544,ZNF776,GTF2B,  GRHL1,HOXB6,ZNF260,ZNF713,YY2,SMAD1,NFE2L2,NFYB,PBX1,ZNF639,ZNF44,ZNF853,ZNF562,NKRF,ZBTB26,RBAK,ZBED5,  ZNF77,GPBP1L1,SIX1,SKIL,SOX9,KLF5,TBX2,TBX6,TERF2,ZBTB14,ZNF10,ZNF16,ZNF19,ZNF22,ZNF23,ZSCAN21,ZNF121,  ZNF224,ZNF226,ZNF343,ZBTB3,ZNF614,SP6,ZNF512,ZC3H8,ZNF514,YBX3,ZNF766,ZNF625,ZNF765,ZNF561,TBPL1,ZBTB39 |
| Negative correlation  with MOMP Activity in scRNA-Seq cohorts | PCSK1N,MTRNR2L8,AHI1,MTRNR2L1,CCNL2,CLU,PIGR,LUC7L3,DST,SOX6,BPIFB1,SFRP1,NUPR1,MUC4,FOXO1,CCDC144A,  MGP,TFF3,CALD1,PPP1R16A,GATAD1,CHST9,TRIO,NNAT,ASCL1,KIAA1324,PIP,TNNT1,GOLGA8A,MAGEA4,SCGB1D2,COL27A1,  PRAME,SIX1,S100P,AFMID,GOLGA8B,GPC3,DNAH11,DSCAM,PCDH15,SULT1C4,MGST1,CRISP3,ID4,NPW,PTCH2,MTRNR2L12,  VEGFA,ADGRV1,TNR,TTYH1,KIF12,FGFR1,ATAD3C,EGFR,HPN,C3,ADAMTS6,FAM227A,ASIC4,COL21A1,PKDCC,FRMD4A,ZNF704,GABRE,SERPINE1,AZGP1,ZNF117,PDLIM3,PAXBP1,S100A1,PEX1,FOLR1,SPINK1,NTRK3,PPP1R9A,RUFY3,RHPN1,SNAP25,GRIA2,  RAMP1,SLPI,ANKRD36C,TF,PKD1,FREM1,CSAD,PNISR,PRRT2,WDR60,GALNT13,SCGB3A1,PTCH1,MAMDC4,CHRM3,ELF3,  ANKRD36B,GPT,SLC12A2,NDRG1,LTF,CEACAM1,FTCD,TFF2,TNNI3,SPAG17,CP,PON3,ONECUT2,SPPL2B,TWIST1,SMOC1,TET1,  MYT1L,PLEKHG4B,FHIT,ABCC3,CDHR3,NTRK2,SMIM22,TMEM98,OPHN1,ERBB4,MLXIPL,CACNA1A,MAPK10,MMP16,FGF12,PRR4,LTBP4,TSHZ2,DLGAP1,SETBP1,OBSCN,HPSE2,CGNL1,C19orf33,EPHA6,ARHGEF38,PLAT,CFAP44,FADS2,CHGA,SCN1A,TTC3,  NRXN1,STC2,WFDC2,PLXNA3,CACNA1C,NREP,ERBB3,ZNF471,NFIB,LSAMP,HIF3A,HPX,NAV2,SEZ6L2,KRT15,SYNE4,RAB3B,  ZNF793,SPTBN4,CSMD3,OCLN,SNTG1,SYNGAP1,CXCL14,YJEFN3,SLC28A3,GPC6,KALRN,PPDPF,TMEM45B,LY6K,NEBL,TCN1 |

# Reference:

1. Wolf DM, Yau C, Wulfkuhle J, et al. Redefining breast cancer subtypes to guide treatment prioritization and maximize response: Predictive biomarkers across 10 cancer therapies. *Cancer Cell*. 2022;40(6):609-623.e6. doi:10.1016/j.ccell.2022.05.005

2. Zhao J, Chen AX, Gartrell RD, et al. Immune and genomic correlates of response to anti-PD-1 immunotherapy in glioblastoma. *Nat Med*. 2019;25(3):462-469. doi:10.1038/s41591-019-0349-y

3. Kim ST, Cristescu R, Bass AJ, et al. Comprehensive molecular characterization of clinical responses to PD-1 inhibition in metastatic gastric cancer. *Nat Med*. 2018;24(9):1449-1458. doi:10.1038/s41591-018-0101-z

4. Cho JW, Hong MH, Ha SJ, et al. Genome-wide identification of differentially methylated promoters and enhancers associated with response to anti-PD-1 therapy in non-small cell lung cancer. *Exp Mol Med*. 2020;52(9):1550-1563. doi:10.1038/s12276-020-00493-8

5. Jung H, Kim HS, Kim JY, et al. DNA methylation loss promotes immune evasion of tumours with high mutation and copy number load. *Nat Commun*. 2019;10(1):4278. doi:10.1038/s41467-019-12159-9

6. Braun DA, Hou Y, Bakouny Z, et al. Interplay of somatic alterations and immune infiltration modulates response to PD-1 blockade in advanced clear cell renal cell carcinoma. *Nat Med*. 2020;26(6):909-918. doi:10.1038/s41591-020-0839-y

7. Ascierto ML, McMiller TL, Berger AE, et al. The Intratumoral Balance between Metabolic and Immunologic Gene Expression Is Associated with Anti-PD-1 Response in Patients with Renal Cell Carcinoma. *Cancer Immunol Res*. 2016;4(9):726-733. doi:10.1158/2326-6066.CIR-16-0072

8. Mariathasan S, Turley SJ, Nickles D, et al. TGFβ attenuates tumour response to PD-L1 blockade by contributing to exclusion of T cells. *Nature*. 2018;554(7693):544-548. doi:10.1038/nature25501

9. Rose TL, Weir WH, Mayhew GM, et al. Fibroblast growth factor receptor 3 alterations and response to immune checkpoint inhibition in metastatic urothelial cancer: a real world experience. *Br J Cancer*. 2021;125(9):1251-1260. doi:10.1038/s41416-021-01488-6

10. Auslander N, Zhang G, Lee JS, et al. Robust prediction of response to immune checkpoint blockade therapy in metastatic melanoma. *Nat Med*. 2018;24(10):1545-1549. doi:10.1038/s41591-018-0157-9

11. Hugo W, Zaretsky JM, Sun L, et al. Genomic and Transcriptomic Features of Response to Anti-PD-1 Therapy in Metastatic Melanoma. *Cell*. 2016;165(1):35-44. doi:10.1016/j.cell.2016.02.065

12. Liu D, Schilling B, Liu D, et al. Integrative molecular and clinical modeling of clinical outcomes to PD1 blockade in patients with metastatic melanoma. *Nat Med*. 2019;25(12):1916-1927. doi:10.1038/s41591-019-0654-5

13. Gide TN, Quek C, Menzies AM, et al. Distinct Immune Cell Populations Define Response to Anti-PD-1 Monotherapy and Anti-PD-1/Anti-CTLA-4 Combined Therapy. *Cancer Cell*. 2019;35(2):238-255.e6. doi:10.1016/j.ccell.2019.01.003

14. Riaz N, Havel JJ, Makarov V, et al. Tumor and Microenvironment Evolution during Immunotherapy with Nivolumab. *Cell*. 2017;171(4):934-949.e16. doi:10.1016/j.cell.2017.09.028

15. Van Allen EM, Miao D, Schilling B, et al. Genomic correlates of response to CTLA-4 blockade in metastatic melanoma. *Science*. 2015;350(6257):207-211. doi:10.1126/science.aad0095

16. Hu C, Li T, Xu Y, et al. CellMarker 2.0: an updated database of manually curated cell markers in human/mouse and web tools based on scRNA-seq data. *Nucleic Acids Research*. 2023;51(D1):D870-D876. doi:10.1093/nar/gkac947
